# The amino-terminal tails of histones H2A and H3 coordinate efficient base excision repair, DNA damage signaling and postreplication repair in *Saccharomyces cerevisiae*

**DOI:** 10.1093/nar/gkv372

**Published:** 2015-04-20

**Authors:** Rithy Meas, Michael J. Smerdon, John J. Wyrick

**Affiliations:** School of Molecular Biosciences, Washington State University, Pullman, WA 99164-7520, USA

## Abstract

Histone amino-terminal tails (N-tails) are required for cellular resistance to DNA damaging agents; therefore, we examined the role of histone N-tails in regulating DNA damage response pathways in *Saccharomyces cerevisiae*. Combinatorial deletions reveal that the H2A and H3 N-tails are important for the removal of MMS-induced DNA lesions due to their role in regulating the basal and MMS-induced expression of DNA glycosylase Mag1. Furthermore, overexpression of Mag1 in a mutant lacking the H2A and H3 N-tails rescues base excision repair (BER) activity but not MMS sensitivity. We further show that the H3 N-tail functions in the Rad9/Rad53 DNA damage signaling pathway, but this function does not appear to be the primary cause of MMS sensitivity of the double tailless mutants. Instead, epistasis analyses demonstrate that the tailless H2A/H3 phenotypes are in the *RAD18* epistasis group, which regulates postreplication repair. We observed increased levels of ubiquitylated PCNA and significantly lower mutation frequency in the tailless H2A/H3 mutant, indicating a defect in postreplication repair. In summary, our data identify novel roles of the histone H2A and H3 N-tails in (i) regulating the expression of a critical BER enzyme (Mag1), (ii) supporting efficient DNA damage signaling and (iii) facilitating postreplication repair.

## INTRODUCTION

In human cells, DNA lesions frequently occur due to a variety of endogenous sources including nonenzymatic methylation of nucleosides by S-adenosylmethionine, cytosine deamination to uracil, and base oxidation by reactive oxygen species (1). Left untreated, these DNA adducts can interrupt regular cellular processes such as replication and transcription; therefore, cells coordinate different DNA damage response mechanisms such as DNA damage checkpoints, excision repair and postreplication repair to maintain genomic integrity.

The base excision repair (BER) pathway is essential for the removal of non-helix distorting lesions caused by methylated and oxidized bases. Typically, BER is initiated by recognition of the DNA lesion by a monofunctional DNA glycosylase that cleaves the N-glycosidic bond to release the chemically modified base, creating an apurinic/apyrimidinic (AP) site. The AP site is then recognized by an AP endonuclease that cleaves the DNA backbone 5′ of the AP site. One of two subpathways is then employed to replace the damaged nucleotide by DNA synthesis of a single nucleotide (short patch repair), or a small run of nucleotides (long patch repair), using the undamaged strand as a template. Repair concludes when the two DNA ends are ligated by a DNA ligase (1).

In the presence of high levels of DNA damage, the 9-1-1 clamp (consisting of the Rad17, Mec3 and Ddc1 complex in *Saccharomyces cerevisiae*) and a Mec1-containing complex are recruited to DNA lesions and, in conjunction with the Rad9 checkpoint adaptor protein, stimulate the hyperphosphorylation of the effector kinase Rad53 (2–4). The phosphorylation of Rad53 leads to cell-cycle arrest at G1/S, intra-S or G2/M phases to allow time for excision repair, such as BER, to remove the lesions before reinitiation of the cell cycle (5). Furthermore, Rad53 phosphorylates the effector protein Dun1 to stimulate the expression of various DNA damage responsive genes including the ribonucleotide reductases (RNR) and the BER glycosylase Mag1 (6).

DNA replication is particularly vulnerable to DNA lesions as replicative DNA helicases create ssDNA molecules that cannot be fully processed by BER because they lack the complementary strand to resynthesize the damaged strand. Furthermore, unrepaired lesions and single-strand break repair intermediates can lead to replication fork collapse. To deal with these problems, the cell utilizes two postreplication repair pathways—translesion synthesis (TLS) and damage avoidance—to bypass DNA lesions. TLS incorporates nucleotides across from damaged bases and can either be error-free or error-prone depending on the type of DNA damage and the translesion polymerase (7). The mechanism of damage avoidance is still not well understood, but it is an error-free pathway that is believed to entail the use of an undamaged copy of the sequence as a template. During replication, this would involve using the newly synthesized daughter strand for homology-directed template switching (8).

The DNA damage response in eukaryotic cells requires that multiple pathways must access and act upon DNA lesions that are packaged into chromatin (9). The primary subunit of chromatin is the nucleosome core particle—a complex consisting of ∼147 base pairs of DNA wrapped 1.65 times around a histone octamer comprised of the four canonical core histones: H2A, H2B, H3 and H4 (10,11). Each of these histones contains an unstructured amino-terminal tail (N-tail) that has been shown to facilitate intra- and inter-nucleosomal interactions *in vitro* (12,13). *In vivo*, the combinatorial deletions of H2A/H2B, H3/H4 and H2A/H4 N-tail pairs are lethal, indicating these N-tail pairs are redundant for cell viability (14–16). The N-tails of histones extend out from the nucleosome core and some of these residues are posttranslationally modified in response to DNA damage. For example, it was found that the canonical histones undergo acetylation after UV irradiation exposure in human cells and lysine residues 9 and 14 of H3 appear to be hyperacetylated after UV irradiation in budding yeast (17,18). Additionally, acetylation of H3 has been shown to facilitate recruitment of Swi/Snf chromatin remodeling complexes *in vitro*, which is important for the removal of UV-induced lesions in chromatin (19,20). Furthermore, the Set1 histone methyltransferase, which methylates lysine 4 in the histone H3 N-tail, contributes to the yeast S-phase checkpoint (21). Posttranslational modifications (PTMs) in the histone core domains, such as ubiquitylation at lysine residue 123 of H2B and methylation at lysine residue 79 of H3 are also needed to activate Rad53 phosphorylation and cell cycle arrest after exposure to DNA damaging agents (22). These results point to the importance of histone residues and histone PTMs in regulating the DNA damage response.

To better understand the role of histone N-tails in the DNA damage response, we measured cell survival, damage checkpoint signaling, and BER activity of single N-tail deletions and viable N-tail deletion pairs in response to DNA alkylation damage by MMS. The N-tail deletions show varying degrees of MMS sensitivity suggesting a role in the DNA damage response. We also found that the N-tail of H3 is important for Rad53 phosphorylation after a short exposure to MMS and is in the *RAD9* epistasis group. Interestingly, the combinatorial N-tail deletions of H2A and H3 resulted in a significant BER deficiency; however, the BER defect in this mutant is not the major contributing factor to MMS sensitivity, as enhancing repair activity with additional Mag1 did not rescue the MMS sensitivity phenotype. Subsequent epistasis analysis revealed that the N-tails of H2A and H3 are in the *RAD18* epistasis group, which is integral for postreplication repair. In agreement with our epistasis results, mutagenesis assays indicate that the H2A and H3 N-tails affect spontaneous and MMS-induced mutation frequencies at the *CAN1* locus. Altogether, our data suggest that the N-tails of H2A and H3 function in multiple signaling and repair pathways to coordinate an efficient response to alkylating agents.

## MATERIALS AND METHODS

### Yeast strains and plasmids

Yeast strains used in this study are described in Supplementary Table S1. Specifically, the residues that were deleted in the N-tail mutants are as follow: residues 1–20 of H2A, residues 1–32 of H2B, residues 1–30 of H3, and residues 1–16 of H4 (16). Yeast genes were myc-tagged as previously described (23). The *MAG1* overexpression vectors were constructed by inserting the restriction digested PCR amplicon into the p416 *P_ADH1_* vector containing the *URA3* marker (American Type Culture Collection).

### DNA damage sensitivity assays

Spot assays were performed by growing cells to 1.25 × 10^7^ cells/ml and spotted in 10-fold serial dilutions onto yeast peptone dextrose (YPD) plates with the specified amount of MMS. UV sensitivity assays were performed by exposing the spotted cells to 254 nm light from two low-pressure Hg lamps (Sylvania, Model G30T8). UV doses were measured with a Spectroline DM-254N UV meter (Spectronics Corp., Westbury, NY, USA).

Survival assays were performed by spreading cells onto YPD plates under the specified treatments and were initially incubated at 30°C in a dark room to inhibit photoreactivation for 3–5 days. Colonies were counted and compared to the untreated cells, which were scored as 100% survival. Cells that contain the p416 *P_ADH1_* overexpression vector were grown on synthetic complete (sc)-uracil plates with the indicated amount of MMS.

### Western blot analysis

Cells were grown to mid-log phase and prepared as previously described (24). The antibodies used are as follows: anti-myc (Abcam, ab18185), anti-GAPDH (Thermo, MA5-15738), anti-Rad53 (Santa Cruz, SC-6748), anti-actin (Sigma, A2066), anti-mouse secondary (Bio-Rad Lab, 170-6516), and anti-rabbit secondary (Bio-Rad Lab172-1019). PCNA ubiquitylation was measured by western blot using an anti-myc antibody to detect myc-tagged PCNA. Mono- and di-ubiquitylated PCNA were present as DNA damage-induced higher molecular weight bands that disappeared in a *rad6Δ* mutant.

### Northern blot analysis

Mid-log phase cells were treated with 0.2% (v/v) MMS for 10 min. RNA was extracted by resuspending cells in TES buffer (10 mM Tris–HCl pH 7.5, 10 mM ethylenediaminetetraacetic acid (EDTA), 0.5% sodium dodecyl sulphate (SDS)) and phenol–chloroform–isoamyl alcohol (PCI) pH 4.7 at 65°C for 30 min. Samples were run on an alkaline agarose gel and then transferred to Hybond-N^+^ nylon membranes (GE Healthcare). The membranes were then probed as previously described (25).

### Repair assays

Cells were grown to mid-log phase in yeast YPD medium and arrested with nocodazole at a final concentration of 15 ug/ml before treatment with 0.2% MMS for 10 min. The genomic repair assay and its quantification were done as previously described with minor modifications (26,27). After MMS exposure, cells were washed with double-distilled H_2_O and resuspended in YPD with 15 ug/ml nocodazole. Cells were collected and DNA was extracted as previously described (24). Briefly, the purified DNA (5–10 μg) was treated with or without human DNA glycosylase (AAG, a gift from Dr Leona Samson, Massachusetts Institute of Technology) and AP endonuclease (APE1, New England Biolabs) in reaction buffer (70 mM MOPS, pH 7.51, 1 mM dithiothreitol, 1 mM EDTA and 5% glycerol) for 1 h at 37°C to generate single strand breaks specifically at methylated purines. These fragmented DNA molecules were then resolved on a 1.2% alkaline gel and stained with SYBR Gold (Invitrogen). The gels were scanned with a Typhoon FLA 7000 (GE Healthcare) and analyzed by ImageQuant 5.2 (Molecular Dynamics) to determine the data profile of each lane. The number of methylpurines/kb was determined by calculating the ensemble average of each lane corrected by the no enzyme control lane, as previously described (27).

Repair of DNA in specific chromatin loci was performed as described previously (28). Briefly, after DNA extraction, the DNA was treated with both EcoRV and EcoRI (New England Biolabs) to release a ∼2.2 kb DNA fragment. The sample was then split into two equal aliquots, treated with or without AAG/APE1 and separated on an alkaline gel. The gel was neutralized (1.5 M NaCl, 1 M TrisCl, pH 7.5) and the DNA transferred to a Hybond-N^+^ membrane to be probed by radiolabled probes using the Prime-It Random Primer Kit (Stratagene). Scans were taken on a Typhoon FLA 7000 (GE Healthcare) and analyzed by ImageQuant 5.2 (Molecular Dynamics). The level of repair was calculated from the equation: *μ* = −ln(*P*_0_), where *μ* is the average number of methylpurines within a region and *P*_0_ is the fraction of uncut restriction fragments.

High resolution mapping of methylpurines at the *RPB2* locus was performed as described previously (29,30). Extracted DNA was treated with DraI to release the *RPB2* gene fragment of 1144 bp. An oligonucleotide complementary to the non-transcribed strand (NTS) of *RPB2* containing a string of dT ending with a biotin tag at its 5′ end was then used to pull down the NTS of *RPB2* via streptavidin beads (Invitrogen). The annealed oligonucleotide was used as a template to tag the pulled-down DNA with [^32^P] dA by using the Sequenase Sequencing kit (Affymetrix). The sample was eluted from the beads and separated on a 6% sequencing gel. The gel was dried, exposed to a PhosphoImager screen (Molecular Dynamics), and scanned on a Typhoon FLA 7000. Each lane was analyzed using ImageQuant 5.2 and the overlapping peaks deconvoluted using Peakfit (SPSS Inc.). The analysis was performed as previously described (31).

### Mutagenesis assays

Cells were grown to 1.25 × 10^7^ cells/ml and split into two sets. One set was left untreated and the other set was treated with 0.2% MMS for 40 min followed by a wash with double-distilled H_2_O to remove MMS. Then 1.25 × 10^9^ to 5 × 10^7^ cells were plated onto sc-arginine media containing 50 μg/ml of canavanine. Dilutions of these cells were then plated onto sc media to establish the initial cell count. After 3–5 days incubation at 30°C, colonies were counted via Quantity One software (Bio-Rad). Mutation frequency was determined by comparing growth on sc to sc + canavanine, while survival was determined by comparing growth of the cells treated with MMS to untreated cells.

## RESULTS

### The N-tails of H2A and H3 play redundant roles in excision repair

**Figure 1.**
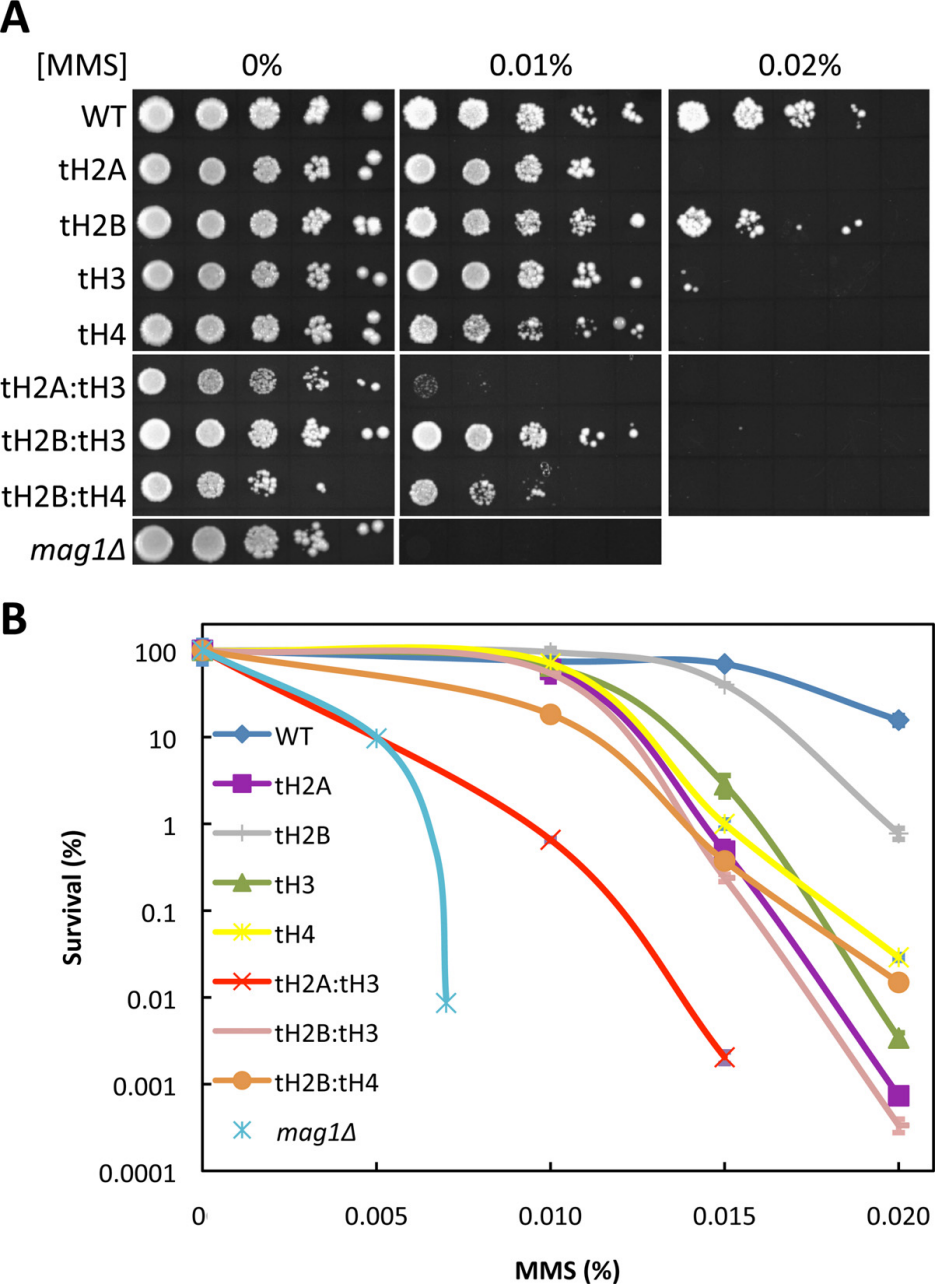
The N-tails of the canonical histones contribute to MMS sensitivity. (**A**) WT and histone N-tail deletion mutants were serially spotted onto YPD plates containing varying concentrations of MMS. A *mag1Δ* mutant is used to show MMS-hypersensitivity. (**B**) Cell survival assay of the tailless histone mutants from (A).

The N-tail deletions of yeast histones have been shown to cause varying degrees of cell sensitivity to some DNA damaging agents as compared to wildtype (WT) cells (16). To analyze sensitivity of the tail-deleted strains (denoted as tH2A, tH2B, tH3, and tH4) to helix distorting lesions, we irradiated yeast cells with UV light, which induces two major photoproducts: *cis–syn* cyclobutane dipyrimidines and pyrimidine-(6-4)-pyrimidone photoproducts. Consistent with previous reports, single or double histone tail deletions led to varying degrees of UV sensitivity, with the sensitivity of the tH2A:tH3 mutant (i.e. H2A Δ1–20 and H3 Δ1–30) being the most severe amongst the N-tail deletion mutants (Supplementary Figure S1) (16,32–33). To determine if a similar phenotype is associated with the tail deletions toward non-helix distorting lesions, we measured cell survival in the presence of the alkylating agent methyl methanesulfonate (MMS) (Figure 1). MMS induces two major methylpurine adducts, 7-methylguanine and 3-methyladenine, that are removed by BER. The tH2A and tH3 single mutants exhibit mild sensitivity to MMS, but cells expressing tH2A and tH3 together show a strong sensitivity to the drug (Figure 1). In comparison, a mutant that lacks the glycosylase that recognizes MMS-induced methylpurines, *mag1Δ*, is more sensitive to MMS than the tH2A:tH3 double mutant (Figure 1A and B).

**Figure 2.**
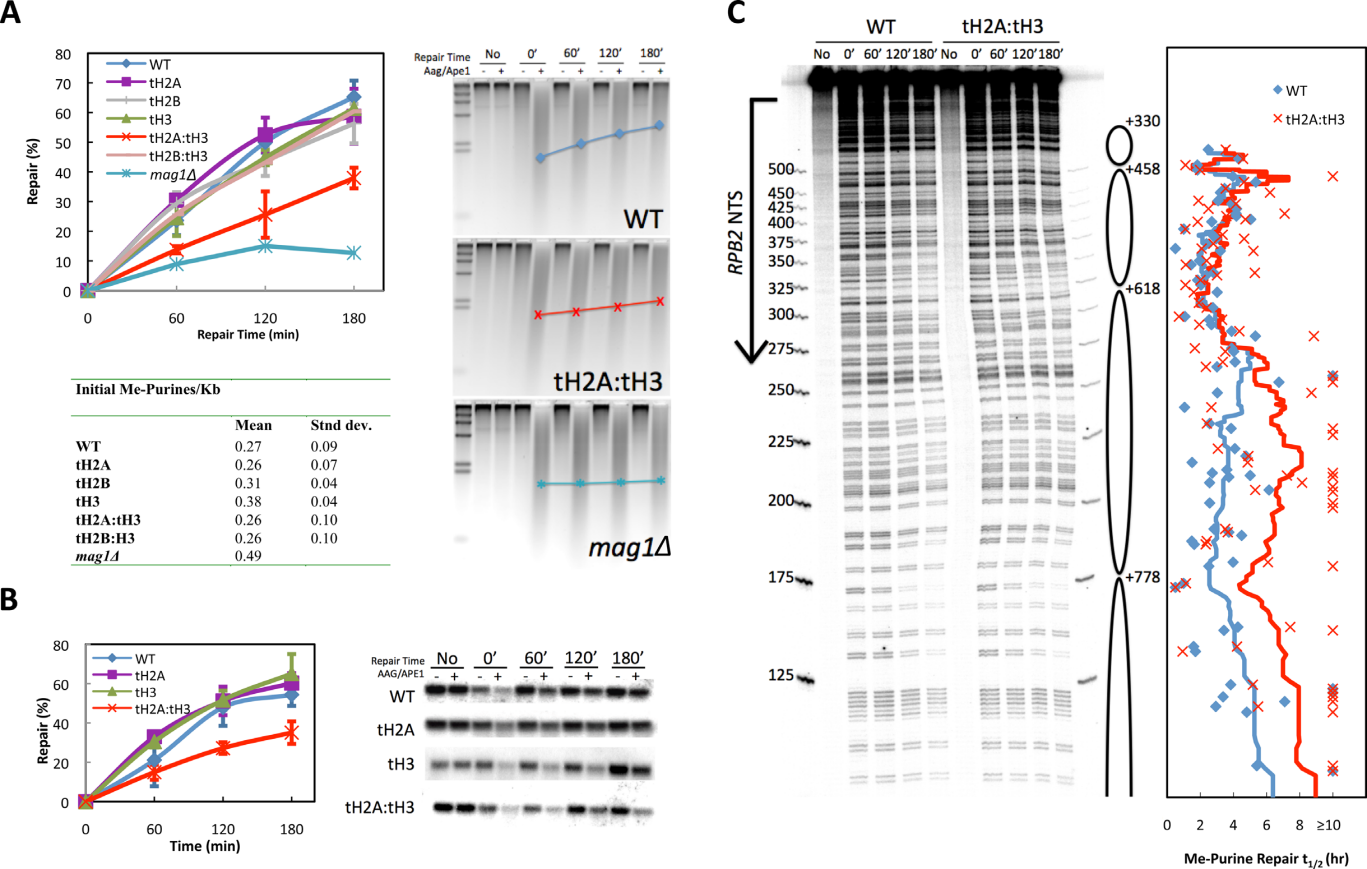
The tH2A:tH3 mutant has reduced repair of methylpurines. In these experiments, the cells were arrested with nocodazole to prevent entry into S-phase, treated with 0.2% MMS for 10 min and then released into YPD containing nocodazole. (**A**) Genomic BER assays were performed on the tailless histone mutants. Representative alkaline gels for WT, tH2A:tH3 and *mag1Δ* cells are shown for DNA samples treated in the absence or presence (+/−) of AAG and APE1, which create single stranded nicks at 3-methyladenine and 7-methylguanine. The average fragment length is denoted for each time point (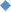, WT; 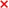, tH2A:tH3; and 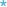, *mag1Δ*). The % of methylpurines removed at each time (or % repair) is shown in the graph as mean ± standard deviation of at least three independent experiments. The initial methylpurines/kb are listed in the table. (**B**) Repair was analyzed at the *GAL10* locus by Southern blotting using locus-specific probes. Graph depicts mean ± standard deviation of at least three independent experiments. (**C**) High resolution DNA damage mapping was performed on the NTS of *RPB2* for WT and tH2A:tH3 cells. The top band on the sequencing gel represents the full-length band, while bands that migrate faster represent methylpurine damage sites that have been cleaved by AAG and APE1 digestion. The arrow on the left indicates the transcription start site and the ovals on the right indicate the most prominent nucleosome positions along *RPB2* as mapped by Jiang *et al*. (34). The plot is aligned so that each point is indicative of the *t*_0.5_ (time to repair 50% of lesions) for its respective band. The smoothed lines were determined by averaging the *t*_0.5_ of 41 nucleotides surround each nucleotide site, where the 41 nucleotides encompass the nucleotide position and 20 nucleotides flanking that nucleotide in each direction.

To determine if the hypersensitivity to MMS shown by the tH2A:tH3 mutant is due to a reduction in BER of alkylated bases in the mutant, we directly measured the overall repair of genomic DNA in the tailless histone strains after MMS treatment in the presence of nocodazole to arrest cells and prevent DNA replication. We found that the single tail deletions of H2A, H2B or H3 result in repair of MMS-induced lesions to a similar degree as that of WT cells in the genome overall (Figure 2A). Importantly, consistent with the MMS sensitivity results, the time course of MMS damage removal is significantly decreased in the tH2A:tH3 double tailless mutant, although repair is still evident (compare tH2A:tH3 mutant to *mag1Δ* in Figure 2A). In contrast, the tH2B:tH3 double tailless mutant had no detectable effect on MMS damage removal, indicating that the histone-associated repair defect is specifically linked with the combinatorial deletion of the tails of H2A and H3. By calculating the ensemble average DNA size on the alkaline gels, we determined the projected average fragment length for each repair time (Figure 2A, gels). As shown in the tH2A:tH3 gel (Figure 2A, middle gel panel), the ensemble average DNA size of the tH2A:tH3 mutant is significantly retarded compared to the WT. Moreover, this defective genomic BER in tH2A:tH3 mutant cells is not due to a difference in the amount of damage associated with the MMS exposure, as the initial levels of methylpurines/kb is similar amongst the various strains (Figure 2A, table).

Since the repair of genome-wide DNA lesions induced by MMS is less efficient in the tH2A:tH3 mutant, we further examined repair at specific chromatin loci that have different levels of transcriptional activity and chromatin organization. We first analyzed repair at the *GAL10* locus in MMS treated cells grown in glucose-containing media, which represses expression of the *GAL10* gene. As found for genomic BER (Figure 2A), the single tail deletions of H2A or H3 did not impair removal of methylpurines at the *GAL10* locus under repressed conditions, whereas the deletion of both tails show defective repair (Figure 2B). We also used a high-resolution DNA damage-mapping assay to analyze repair at the constitutively active *RPB2* locus, which encodes the second largest subunit of RNA polymerase II (30). A *MAG1* deletion mutant (*mag1Δ*) essentially abolishes repair of methylpurine lesions at this locus (30). We found that repair along the NTS of *RPB2* is repressed in the nucleosome core regions (as determined in (34)) of WT and tH2A:tH3 cells (Figure 2C). Furthermore, quantitative analyses of the band intensities indicate that, overall, the *t*_0.5_ (or time required to repair half the lesions) was extended in the tH2A:tH3 mutant cells.

Collectively, these data show that efficient BER of MMS-induced lesions is dependent on both the H2A and H3 N-terminal tails. Not surprisingly, when we analyzed nucleotide excision repair of UV-induced lesions in the genome overall, we also see a small, but significant, decrease in repair in the tH2A:tH3 mutant, indicating the importance of the tails for both excision repair pathways (Supplementary Figure S2).

### The N-tails of H2A and H3 are important for proper expression of repair proteins

**Figure 3.**
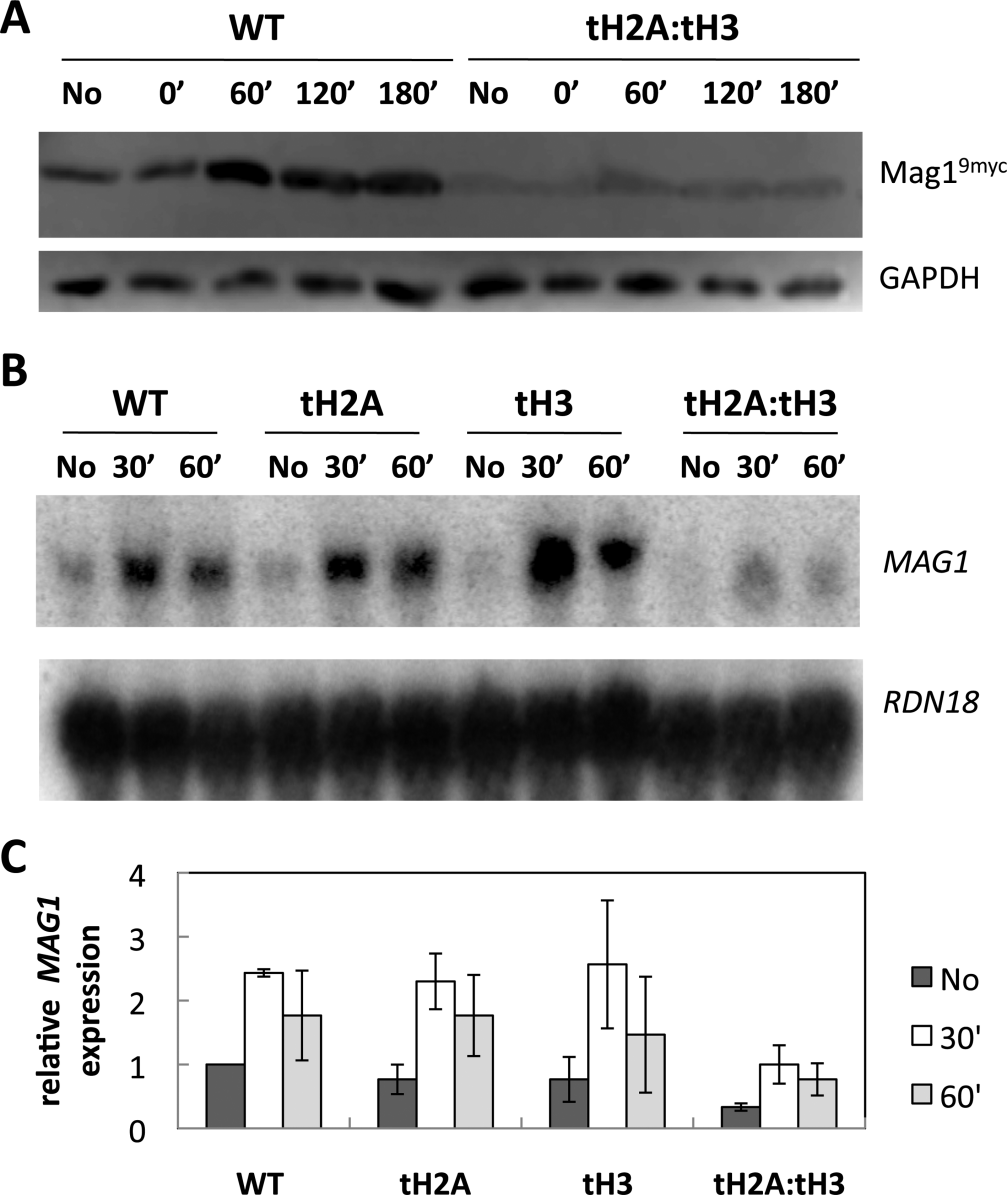
Mag1 protein expression and *MAG1* mRNA expression are reduced in tH2A:tH3 cells. WT and the N-tail deletion mutants were treated with 0.2% MMS for 10 min and further analyzed at the indicated time points. (**A**) Mag1^9myc^ and GAPDH were detected via western blotting. (**B**) A northern blot detecting *MAG1* and reprobed for *RDN18*. [We note that the small band shift between the tH3(60’) sample and the tH2A:tH3 samples is due to a gel running artifact]. (**C**) Quantification of different gels, such as shown in (B) by normalizing *MAG1* levels to that of WT in the absence of MMS. Error bars indicate standard deviations derived from three independent experiments.

Because there is reduced BER activity in tH2A:tH3 mutant cells, we explored the possibility that these cells may have differential expression of BER proteins. Therefore, we analyzed the protein levels of Mag1, the DNA glycosylase that recognizes MMS-induced lesions, and Apn1, the major AP endonuclease in *S. cerevisiae*. Using strains with myc-tagged Mag1 and Apn1, WT and tailless mutants were treated with 0.2% MMS for 10 min and relative protein levels were determined before and 60 min after MMS exposure. In both the WT and single tail deletions of H2A and H3, Mag1 protein levels increased after 60 min; however, this induction and the overall Mag1^9myc^ protein levels are reduced in the tH2A:tH3 mutant (Figure 3A and Supplementary Figure S3A). On the other hand, analysis of Apn1^9myc^ after MMS exposure shows no substantial decrease in the AP endonuclease protein levels in the tH2A:tH3 mutant (Supplementary Figure S3B). To determine if the reduced levels of Mag1 are due to a transcriptional defect, we analyzed the transcription of *MAG1* (compared to *RDN18*, the 18S rRNA) using northern blot analysis. As shown in Figure 3B and C, the basal and MMS-induced mRNA levels of *MAG1* are reduced in the tH2A:tH3 cells (relative to WT), although MMS induction of *MAG1* mRNA still occurs in these cells. This suggests that the reduced Mag1 protein expression in tH2A:tH3 cells is due to a transcriptional deficiency.

There is the possibility that loss of the N-tails of H2A and H3 affects the expression of other repair proteins; therefore, we also analyzed the expression of Mgt1, which is an O^6^-methylguanine-DNA methyltransferase that functions in the direct enzymatic reversal of O^6^-methylguanines. Interestingly, we also observe a decrease in Mgt1 protein levels in the tH2A:tH3 mutant (Supplementary Figure S4). However, our preliminary data indicate that this change in protein level is not due to a transcription defect (data not shown). Taken together, these data indicate that the N-tails of H2A and H3 are important for coordinating the proper expression of repair proteins that respond to alkylated base lesions.

### Overexpression of *MAG1* increases repair but does not rescue the MMS sensitivity phenotype

**Figure 4.**
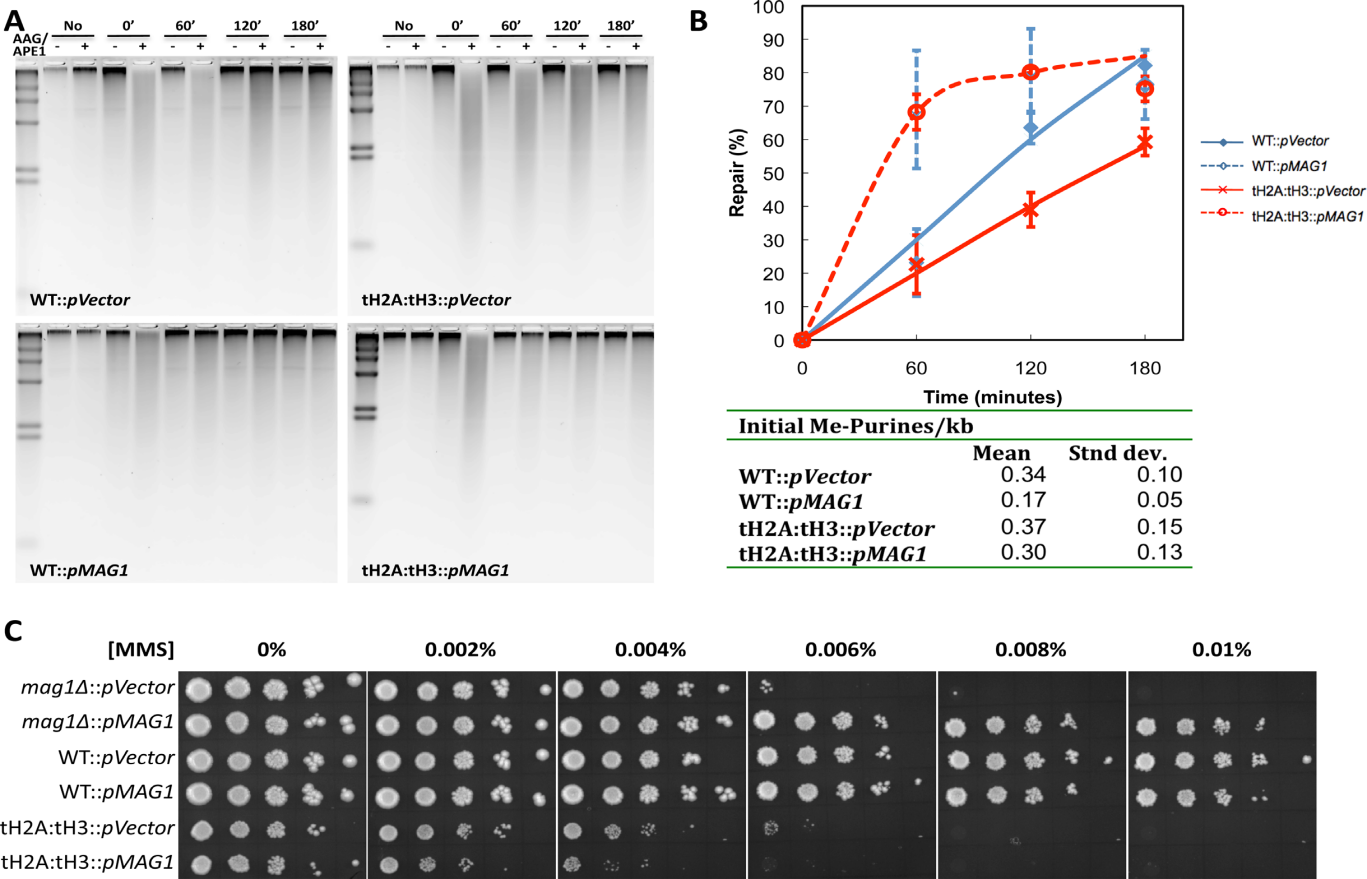
Overexpression of *MAG1* enhances repair but does not suppress the MMS sensitivity in the tH2A:tH3 mutant. (**A**) Representative alkaline gels analyzing BER in genomic DNA were performed similarly to those in Figure 2. (**B**) The quantification of different gels, such as shown in (A), and the initial methylpurines/kb are shown in the table. (**C**) The vector only control (p*Vector*) or the constitutively expressed *MAG1* vector (p*MAG1*) was transformed into *mag1Δ*, WT and tH2A:tH3 cells and serially spotted onto sc-ura plates containing different MMS concentrations.

Since the tH2A:tH3 mutant shows reduced BER activity concomitant with reduced levels of Mag1 protein, we wondered if overexpression of *MAG1* could rescue the MMS sensitivity and repair defects in tH2A:tH3 mutant cells. To test this possibility, we created a vector with *MAG1* under control of the constitutive *ADH1* promoter (p*MAG1*). Using the genomic BER assay to measure removal of MMS-induced lesions, we found that the vector-only controls (p*Vector*) had no effect on repair efficiency in the WT and tH2A:tH3 cells (Figure 4A, upper gels, and B, solid lines). On the other hand, overexpression of *MAG1* in either WT or tH2A:tH3 cells enhanced repair of MMS-induced lesions as compared to the vector only strains, where most of the lesions are repaired within the first 60 min (Figure 4A, lower gels, and B, dashed lines). Furthermore, there appears to be a decrease in the initial level of methylpurines/kb in the WT strain that overexpress *MAG1*, which is most likely due to repair occurring during MMS exposure (Figure 4B, table). Interestingly, this decreased initial level of methylpurines is not seen in the tH2A:tH3::p*MAG1* overexpression strain, which is likely due to the somewhat lower levels of *MAG1* expression in this strain (Supplementary Figure S5, compare WT::p*MAG1* and tH2A:tH3::p*MAG1*). Overall, these data indicate that overexpression of Mag1 results in enhanced BER and the repair defect can be rescued in the tH2A:tH3 mutant.

Next, we examined whether the overexpression of *MAG1* can suppress the MMS sensitivity of tH2A:tH3 mutant cells (Figure 1). The p*MAG1* vector clearly provides functional Mag1 protein, as the expression vector is able to suppress the MMS sensitivity phenotype in the *mag1Δ* mutant (Figure 4C). In fact, the *mag1Δ*::p*MAG1* cells mirror the WT::p*MAG1* strain, which itself shows a slight sensitivity toward MMS that is most likely due to the accumulation of cytotoxic abasic sites, as previously described for these cells (35,36). Surprisingly, the overexpression of *MAG1* did not suppress the MMS sensitivity of the tH2A:tH3 mutant (Figure 4C) even though BER efficiency and *MAG1* transcript levels in the double tailless mutant are greater than WT::p*Vector* (Figure 4B and Supplementary Figure S5). Indeed, like the WT strain overexpressing Mag1, the tH2A:tH3::p*MAG1* is more sensitive to MMS than tH2A:tH3::p*Vector* cells. Thus, the MMS sensitivity of the tH2A:tH3 mutant is not solely due to defective BER caused by reduced expression of Mag1.

### The N-tail of H3 contributes to DNA damage checkpoint signaling

**Figure 5.**
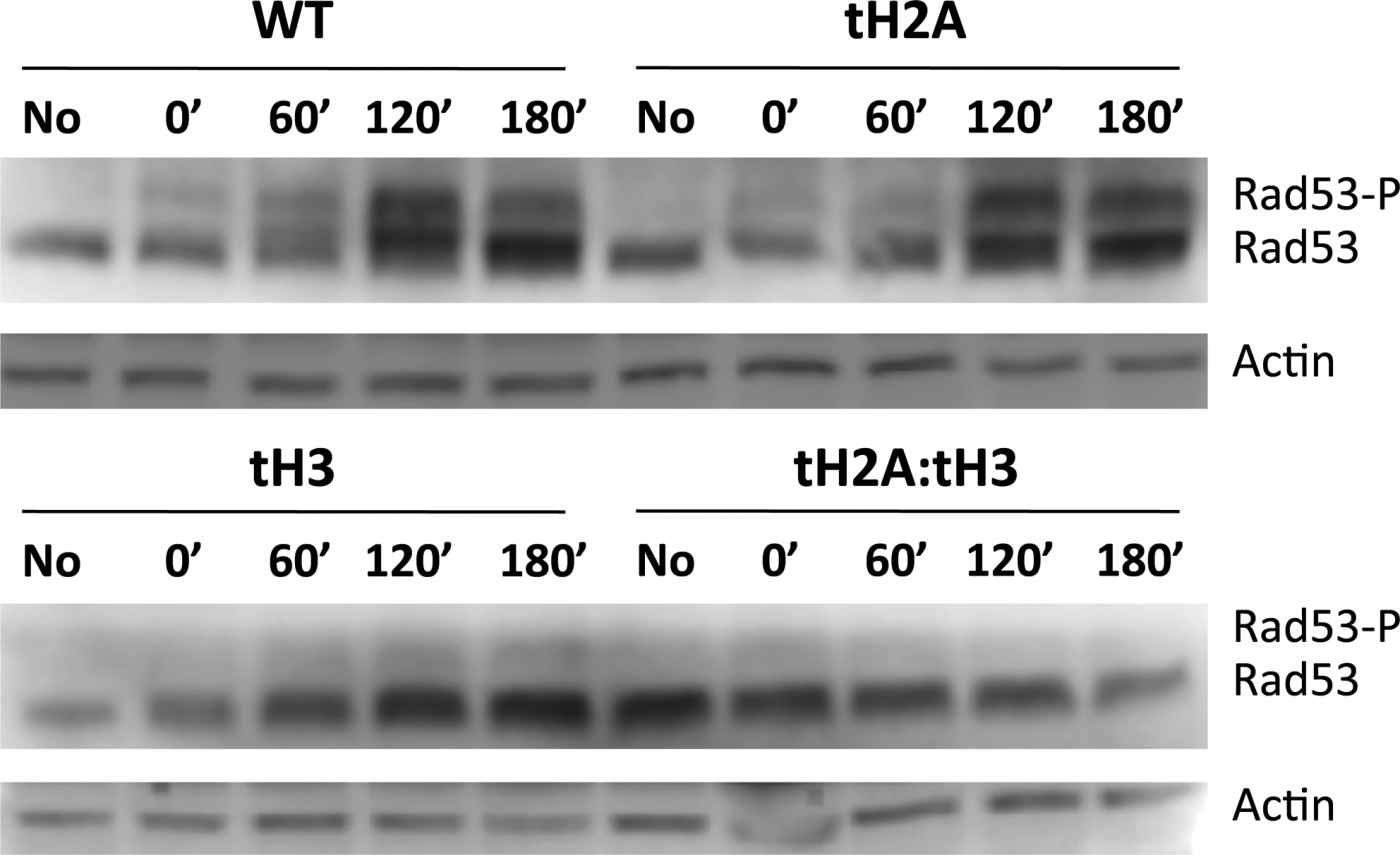
The N-tail of H3 is important for proper Rad53 phosphorylation after DNA damage. Western blot detecting Rad53 and its hyperphosphorylated form (Rad53-P) before and after treatment with 0.2% MMS for 10 min.

We have shown that the N-terminal tails of H2A and H3 are important for efficient BER by modulating the expression of repair proteins; however, enhancing BER by overexpressing *MAG1* does not diminish MMS sensitivity in the tH2A:tH3 mutant. There is the possibility that the MMS sensitivity phenotype is due to the inability of the tH2A:tH3 mutant to properly control checkpoint signaling and cell cycle arrest after DNA damage to facilitate repair. To address this question, we examined phosphorylation of the DNA damage checkpoint effector kinase Rad53. MMS induces phosphorylation of over 15 sites on Rad53 to trigger cell cycle arrest and other DNA damage responses including gene expression changes (4,37–38). Hyperphosphorylation of Rad53 results in a band shift that can be seen after 1 hour of MMS exposure to WT and tailless mutant cells (Supplementary Figure S6A). However, following a 10 minute pulse of 0.2% MMS to the cells, similar to what was used in the repair assays, Rad53 phosphorylation in the tH3 and tH2A:tH3 mutants is impaired (Figure 5). These data indicate that the N-tail of H3, but not of H2A, is important for Rad53 phosphorylation following a short MMS exposure, as opposed to a continuous MMS exposure (compare Figure 5 and Supplementary Figure S6B).

### The N-tails of H2A and H3 are linked to the *RAD18* epistasis group

**Figure 6.**
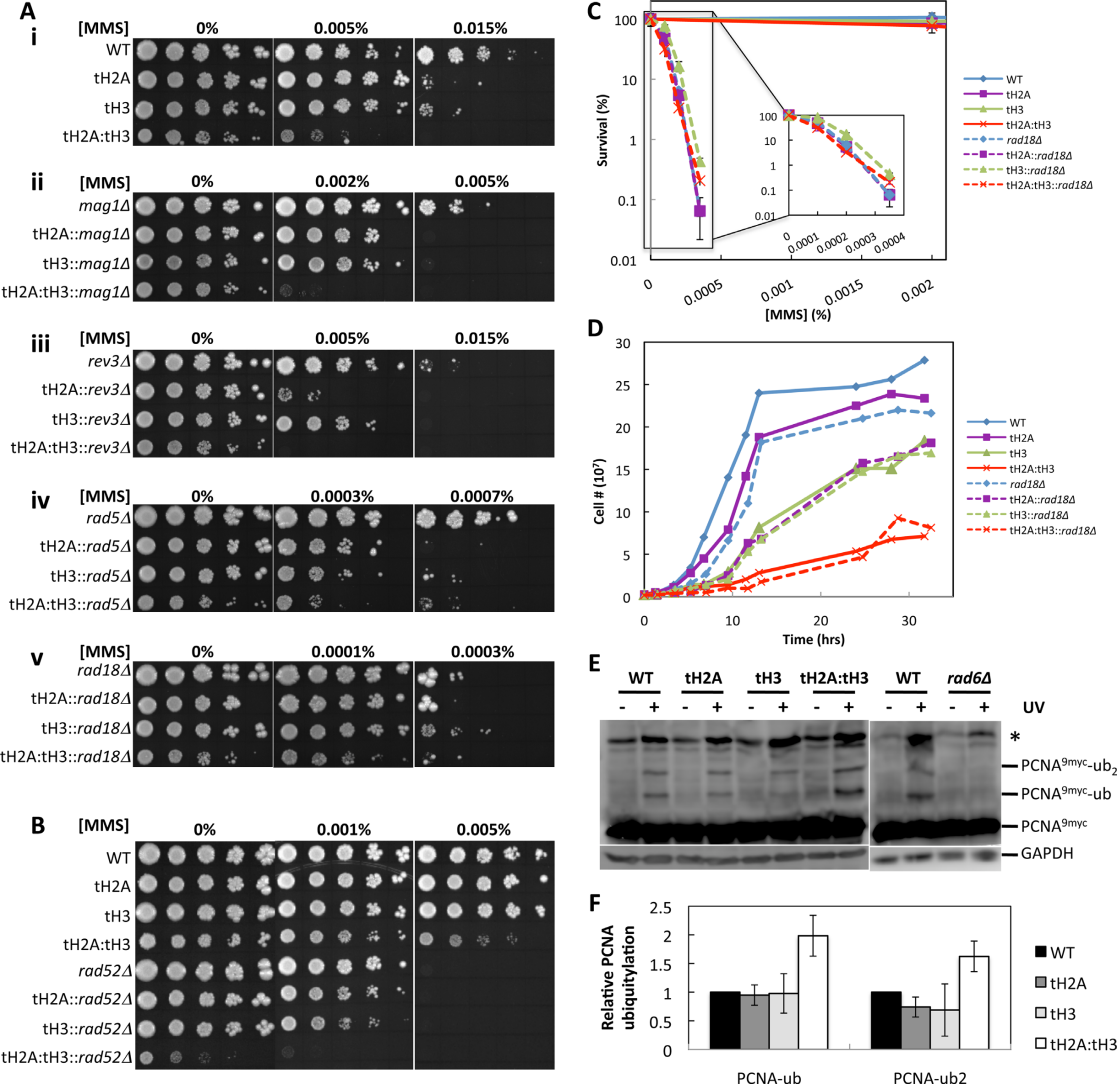
The N-tails of H2A and H3 are epistatic to *RAD18*. (**A**) Epistasis studies of (i) N-tail deletion mutants combinatorially deleted for (ii) *MAG1*, (iii) *REV3*, (iv) *RAD5* and (v) *RAD18*. (**B**) Epistasis study of N-tail deletion mutants combinatorially deleted for *RAD52*. (**C**) MMS survival assay of WT and N-tail mutants (solid lines) and the N-tail mutants combinatorially deleted for *RAD18* (dashed lines). (**D**) Growth curves of the cells in (**C**) in the absence of MMS. (**E**) Western blot of PCNA^9myc^ in WT and N-tail mutants before UV exposure and after a 1 h recovery in YPD following 100 J/m^2^ of UV irradiation. GAPDH is used as the loading control. *rad6Δ* serves as a negative control for PCNA ubiquitylation. (**F**) Quantifications of (E) by normalizing (PCNA^9myc^-ub)/(PCNA^9myc^-total) and (PCNA^9myc^-ub_2_)/(PCNA^9myc^-total) to WT. Error bars indicate standard deviations from three independent experiments.

We performed epistasis studies to genetically identify which DNA damage response pathways are important for the MMS sensitivity of the tH2A:tH3 mutant. First, we tested epistasis for the BER pathway by examining double mutants in which the *MAG1* gene was deleted in the tH2A, tH3, and tH2A:tH3 mutant strains. As shown in Figure 6A.ii, the deletion of *MAG1* with any of the tail deletion combinations showed increased MMS sensitivity over that of the *mag1Δ* mutant alone, indicating that histone tailless mutants are not epistatic to the BER pathway. These data confirm that BER is not the only MMS-response pathway affected when the N-tails of H2A and H3 are deleted.

Since deletion of the H3 N-tail caused misregulation of Rad53 phosphorylation after MMS damage (Figure 5), we wanted to genetically explore the epistatic relationship of the histone N-tails and DNA damage checkpoint control. Rad53 is essential for cell survival; therefore, we opted to delete *RAD9*, a DNA damage adaptor protein that facilitates Rad53 hyperphosphorylation in response to DNA damage (39–41). We found that the single N-tail deletion of histone H3 is just as sensitive to MMS as a double deletion of *RAD9* and the N-tail of H3 (Supplementary Figure S7). To see if the N-tails of H2A and H3 are epistatic with other DNA damage checkpoint pathways, we performed an epistasis analysis with *RAD24*, which functions to facilitate loading of the yeast 9-1-1 complex onto DNA in response to DNA damage (1). We found that the N-tails of H2A and H3 are not epistatic with *RAD24* (Supplementary Figure S8A). These data show that the defect in Rad53 phosphorylation and signaling through the *RAD9* pathway most likely explains the MMS sensitivity of the tH3 single tailless mutant but not the tH3:tH2A double tailless mutant (Figures 2A and 5).

We further explored the role of histone tails in maintaining MMS resistance in additional DNA damage tolerance pathways involved in postreplication repair: TLS and DNA damage avoidance. These postreplication repair pathways are important for cellular survival by allowing polymerase progression through damaged DNA, thus avoiding lethal replication fork collapse (8). To analyze the TLS pathway, we deleted the catalytic component of DNA polymerase ζ, Rev3, which is a key lesion bypass polymerase that has low fidelity when incorporating nucleotides with a damaged DNA template. The concomitant deletion of the H2A and H3 N-tails with *REV3* resulted in enhanced MMS sensitivity as compared to a *REV3* single deletion mutant (Figure 6A.iii), indicating the tail deletions are not epistatic to DNA polymerase ζ. In addition, we tested the two other TLS polymerases—the deoxycytidyl transferase Rev1 and polymerase η (Rad30)—and found that neither is epistatic to the single or double deletions of the H2A and H3 N-tails (Supplementary Figure S8C and S8D). We also analyzed the damage avoidance pathway by deleting Rad5, an enzyme that works together with Ubc13-Mms2 to polyubiquitylate proliferating cell nuclear antigen (PCNA) to allow for homology-directed template switching (8). Similar to the results obtained for *mag1Δ* and *rev3Δ*, the combinatorial deletion of the histone tails and *RAD5* result in enhanced MMS sensitivity over the single deletion mutants (Figure 6A.iv). Further epistasis analysis with *MMS2* confirmed that the N-tails of H2A and H3 are not epistatic to the DNA damage avoidance pathway (Supplementary Figure S8B).

The data in Figure 6A i–iv indicate that either (1) the N-tails of H2A and H3 are not involved in either the BER or postreplicative repair pathways or (2) both tails are involved in multiple DNA damage response pathways. To test these hypotheses, we performed epistasis studies with *RAD6* and *RAD18*, which coordinate DNA damage checkpoint, BER and postreplication repair (8,42–43). Rad6 is an E2 ubiquitin conjugating enzyme that interacts with four known E3 ligases (Rad18, Bre1, Ubr1 and Ubr2) to ubiquitylate specific substrates. Deletion of *RAD6* with either tH2A or tH3 is viable; however, deletion of *RAD6* and both tails of H2A and H3 is synthetically lethal (Supplementary Figure S9). Because Rad6 is known to interact with three different E3 ligases, we deleted each of them to see if one of the four can possibly be synthetically lethal with tH2A:tH3. Each E3 ligase promotes the ubiquitylation of distinct substrates: Bre1 ubiquitylates H2B (44,45), Rad18 ubiquitylates PCNA (46,47), Ubr1 promotes the N-end rule protein degradation pathway (48), and Ubr2 ubiquitylates the proteasomal transcription factor Rpn4 (49). Interestingly, the combinatorial deletion of each E3 ligase with tH2A:tH3 did not lead to a synthetic lethal phenotype (data not shown). This indicates that cell viability is dependent on multiple Rad6 E3 ligases in the tH2A:tH3 mutant.

Because the tH2A:tH3::*rad18Δ* mutant is viable, we tested the epistatic relationship between *RAD18* and the H2A and H3 N-tails. We found that the MMS sensitivity of the *rad18Δ* mutant is not affected by either the single or combinatorial deletions of the H2A and H3 N-tails (Figure 6A.v and C, see overlap of dashed-lined survival curves), indicating that *rad18* is epistatic to the N-tails of H2A and H3. Additionally, both the *rad18Δ* and the H3 N-tail deletion have slow growth phenotypes in normal growth conditions. Hence, we also tested for epistasis using the slow growth phenotype. We found that the growth phenotypes of tH3 and tH2A:tH3 mutants were not affected by the *RAD18* deletion (Figure 6D, compare solid versus dashed green and red lines). These data indicate that the tH3 and tH2A:tH3 mutants are epistatic with *rad18* for cell growth, and the N-tail deletions of H2A and H3 are in the *RAD18* epistasis group to maintain MMS resistance. Intriguingly, like *rad18*, the tH2A:tH3 mutant has a synthetic growth defect when *RAD52* (which is required for homologous recombination) is deleted (Figure 6B, compare the 0% spots of tH2A:tH3::*rad52Δ* to WT and *rad52Δ*) (50). As expected, *rad52* is not epistatic to the N-tails of H2A and H3 (Figure 6B).

Rad18 is an E3 ligase that monoubiquitylates PCNA during DNA damage response to promote postreplication repair pathways; therefore, we asked if the monoubiquitylation of PCNA is affected in the tH2A:tH3 mutant. To answer this question, we myc-tagged PCNA and treated the cells with either UV or MMS and measured PCNA ubiquitylation by western blot. Treatment with 0.1% MMS for 1 h, however, led to little if any detectable DNA damage-induced PCNA ubiquitylation (data not shown). However, we consistently observed a higher level of PCNA mono- and di-ubiquitylation in the tH2A:tH3 mutant after UV treatment (Figure 6E and F).

Members of the *RAD6-RAD18* epistasis group are required for spontaneous and DNA damage-induced mutagenesis (51). Therefore, we analyzed the mutation frequency of the arginine permease *CAN1* in the tailless histone mutants by scoring resistance to canavanine, an analog of arginine that is toxic to the cell when incorporated into proteins (52). As shown in Table 1, our results indicate that the N-tail of H2A does not show a mutagenesis defect when compared to WT. However, consistent with our epistasis studies with *rad18* (Figure 6A.v and C), the *CAN1* mutation frequency decreases in the tH3 mutant. The *CAN1* mutation frequency in the tH2A:tH3 mutant was very low and similar to that of a *rev3* mutant (Table 1), in agreement with our epistasis studies linking the H2A and H3 N-tails with the *RAD18* pathway.

**Table 1.** Mutagenesis frequency of tailless histone mutants

Strain	MMS	*CAN1* mutation frequency (10^−6^)	Survival (%)
WT	−	7.2 ± 0.7	
	+	33.8 ± 1.2	67.3 ± 2.4
tH2A	−	8.7 ± 0.4	
	+	33.1 ± 4.4	52.5 ± 2.5
tH3	−	0.2 ± 0.02	
	+	25.8 ± 1.5	8.1 ± 0.2
tH2A:tH3	−	0.01 ± 0.001	
	+	0.4 ± 0.07	2.3 ± 0.5
*rev3Δ*	−	0.3 ± 0.2	
	+	0.3 ± 0.05	11.1 ± 0.5

## DISCUSSION

**Figure 7.**
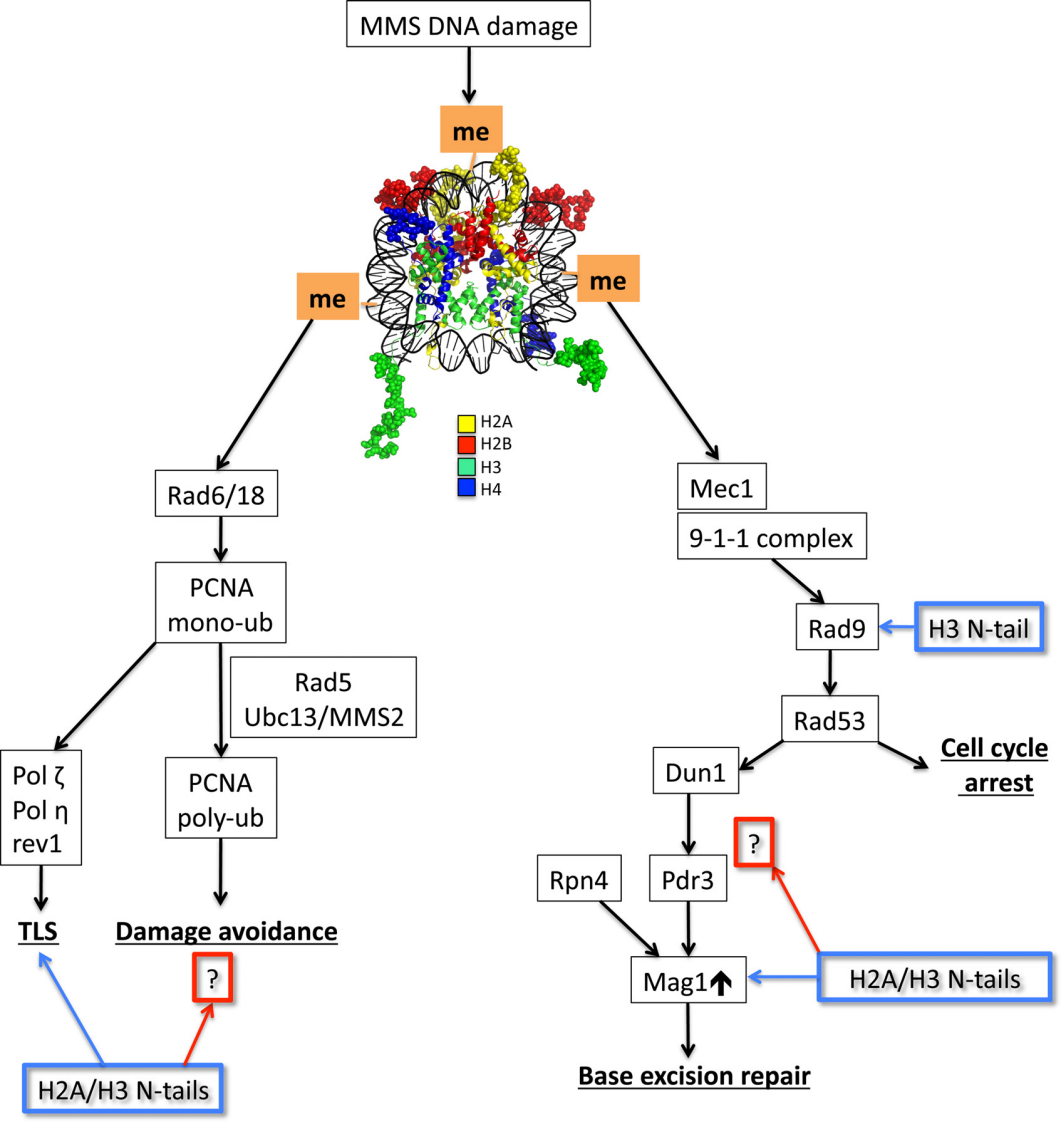
A model highlighting the contributions of H2A and H3 N-tails through different branches of MMS DNA tolerance. The N-tails of all histones within the nucleosome core particle (pdb: 1KX5; MacPyMOL) are represented in the ‘spheres’ configuration. The H3 N-tail is important for the regulation of DNA damage cell cycle checkpoint signaling via Rad9, which facilitates the subsequent hyperphosphorylation of Rad53. H2A and H3 N-tails are also important for upregulating BER gene expression by influencing unknown effectors (?) downstream of Rad53 to regulate Mag1. Furthermore, our epistasis and mutagenesis studies indicate the inclusion of the N-tails of H2A and H3 in the Rad18 epistasis group by influencing postreplication repair through TLS and possibly damage avoidance (?).

Eukaryotic cells respond to DNA damage by activating multiple pathways to promote lesion clearance and cell survival. These pathways must detect and operate on DNA lesions that are packaged in chromatin; however, the effects of chromatin on the DNA damage response are still being elucidated. In this study, we characterized the contributions of the N-tails of canonical histones in the DNA damage response to the alkylating agent MMS in *S. cerevisiae*, an important model system for studying histone function. We have found that the H2A and H3 N-tails have important and unexpected roles in coordinating multiple DNA damage response pathways. The specific functions of the histone N-tails in BER, DNA damage checkpoint signaling, and postreplication repair pathways are summarized in Figure 7 and detailed below.

We found that the tH2A:tH3 mutant is more sensitive to the alkylating agent MMS than the other double tailless mutants or the single tail deletions individually (Figure 1). We initially hypothesized that the MMS sensitivity in the tH2A:tH3 mutant was due to a defect in BER, a repair pathway that excises MMS-induced lesions. BER was analyzed via several repair assays that collectively indicate a global BER deficiency in the tH2A:tH3 mutant (Figure 2). In contrast, single histone tail deletions did not significantly affect BER. Subsequent experiments indicate that the H2A and H3 N-tails are specifically required for the basal and activated expression of the DNA glycosylase Mag1 (Figure 3A–C and Supplementary Figure S3A). Overexpression of Mag1 in the tH2A:tH3 mutant restored efficient BER, confirming that the histone N-tails function in the BER pathway by regulating the expression of this critical BER enzyme (Figure 4A and B).

*MAG1* is one of more than 300 genes that are induced after MMS exposure in budding yeast (53). *MAG1* induction requires the pleiotropic drug resistance protein Pdr3, which bind to an upstream activating sequence in the *MAG1* promoter, and the proteasomal transcription factor Rpn4 (54–56). Currently, it is not clear whether the N-tails of H2A and H3 regulate *MAG1* expression through these transcription factors or through another mechanism. One intriguing possibility is that DNA damage responsive posttranslational modifications of the histone tails may directly modulate *MAG1* expression. The H2A and H3 N-tails are also required for the basal expression of the O^6^-methylguanine DNA methyltransferase Mgt1 (Supplementary Figure S4), which catalyzes the direct enzymatic removal of the MMS-induced O^6^-methylguanine lesion. The *MGT1* promoter shares a common upstream regulatory sequence element with the *MAG1* promoter (57); however, we did not observe a significant decrease in *MGT1* mRNA levels in the tH2A:tH3 mutant. The expression of other repair enzymes, including the *NTG1* and *NTG2* DNA glycosylases and the NER protein Rad23, may be regulated in a similar manner as *MAG1* (58). It will be interesting to determine whether the expression of these genes is also regulated by the H2A and H3 N-tails.

We found that induction of Rad53 phosphorylation in response to MMS damage is compromised in the tH2A:tH3 mutant strain. Our data indicate that the H3 N-tail specifically regulates Rad53 signaling as the Rad53 phosphorylation phenotype of the tH3 mutant is the same as the tH2A:tH3 double mutant (Figure 5). This signaling defect can be suppressed by longer exposures to MMS, indicating the DNA damage checkpoint(s) are only partially compromised in the tH3 mutant (Supplementary Figure S6). Previous studies have shown that H3 K79 methylation and H2A phosphorylation are required for Rad9 chromatin association and Rad53 phosphorylation (21,59). It seems likely that posttranslational modifications in the H3 N-tail (e.g. H3 K4 methylation) are also required for Rad53 activation.

Because Rad53 is required for induction of *MAG1* expression ((60); Figure 7), a defect in Rad53 signaling could explain the observed decrease in *MAG1* expression in the tH2A:tH3 mutant. We do not favor this model for two reasons: (1) *MAG1* expression is not affected in the tH3 mutant even though it has a similar effect on Rad53 phosphorylation as the tH2A:tH3 mutant and (2) the expression of other proteins regulated by the Rad53 pathway (e.g. Rnr2) is not affected in the tH2A:tH3 mutant (data not shown). We favor the model that the N-tails of histones H2A and H3 directly regulate the expression of *MAG1* and potentially other genes.

While overexpression of Mag1 rescued the repair deficiency of the tH2A:tH3 mutant, it did not rescue its MMS sensitivity phenotype (Figure 4). Instead, our epistasis studies indicate that the N-tails of H2A and H3 are in the *RAD18* epistasis group (Figure 6). The Rad6 E2 and Rad18 E3 ubiquitin ligases initiate postreplication repair by ubiquitylating PCNA in response to DNA damage. However, we do not observe a defect in PCNA ubiquitylation in the tH2A:tH3 mutant; to the contrary, we find a significant increase in mono- and polyubiquitylation of PCNA after DNA damage (Figure 6E and F). This could reflect a general increase in replication stress in the tH2A:tH3 mutant. An alternative model is that the increase in PCNA ubiquitylation may reflect a defect in the recruitment or activity of the Ubp10 deubiquitylase, which is required to deubiquitylate PCNA (61). A third possibility that we favor is that completion of postreplication repair is hampered in the tH2A:tH3 mutant, leading to sustained PCNA ubiquitylation by Rad6 and Rad18. This model would also fit the epistasis results indicating that the H2A and H3 N-tails function in postreplication repair.

The *RAD6-RAD18* epistasis group coordinates almost all DNA damage-induced mutations and at least 50% of spontaneous mutations (51). Our mutagenesis assay (Table 1) shows both DNA damage-induced and spontaneous mutagenesis of *CAN1* are reduced in the tH3 and tH2A:tH3 mutants. This further validates that the N-tails of H2A and H3 are in the *RAD18* epistasis group. We hypothesize that the H2A and H3 tails affect the recruitment or activity of TLS polymerases, the main contributors of damage-induced and spontaneous mutagenesis. In summary, multiple lines of evidence support our model that postreplication repair is hampered in the tH2A:tH3 mutant.

Rad18 is required for both branches of postreplication repair: translesion synthesis and error-free damage avoidance. Our epistasis results indicate that the N-tails of H2A and H3 are likely affecting both branches of postreplication repair (Figure 7), since the N-tails are not epistatic with mutants in either branch alone (i.e. Mms2, Rev3, Rad30 and Rev1). It would be of significant interest to determine the exact role of the histone tails in these postreplication repair pathways and their roles in translesion synthesis and mutagenesis. Furthermore, it will be important to characterize which residues or domains within the H2A and H3 N-tails function in this pathway. Interestingly, previous studies have demonstrated that histone N-tails, particularly the H3 N-tail, can be ‘clipped’ by nuclear-localized proteases during the processes of mouse embryonic stem cell differentiation (62) and yeast sporulation (63). It would be intriguing to determine if histone N-tail proteolysis is also a mechanism for regulating postreplication repair pathways and other aspects of the DNA damage response.

## SUPPLEMENTARY DATA

Supplementary Data are available at NAR Online.

SUPPLEMENTARY DATA
