# Supplementary material for: The amino-terminal tails of histones H2A and H3 coordinate efficient base excision repair, DNA damage signaling and postreplication repair in Saccharomyces cerevisiae
Source: Nucleic Acids Res. 2015 Apr 20;43(10):4990–5001. doi: 10.1093/nar/gkv372 (PMC4446432; doi:10.1093/nar/gkv372)
Supplement: SUPPLEMENTARY DATA [file supp_43_10_4990__index.html]

The amino-terminal tails of histones H2A and H3 coordinate efficient base excision repair, DNA damage signaling and postreplication repair in Saccharomyces cerevisiae — The amino-terminal tails of histones H2A and H3 coordinate efficient base excision repair, DNA damage signaling and postreplication repair in Saccharomyces cerevisiae — SUPPLEMENTARY DATA 

# The amino-terminal tails of histones H2A and H3 coordinate efficient base excision repair, DNA damage signaling and postreplication repair in *Saccharomyces cerevisiae*

## SUPPLEMENTARY DATA

**Files in this Data Supplement:**

- SUPPLEMENTARY DATA
