# Supplementary material for: The amino-terminal tails of histones H2A and H3 coordinate efficient base excision repair, DNA damage signaling and postreplication repair in Saccharomyces cerevisiae
Source: Nucleic Acids Res. 2015 Apr 20;43(10):4990–5001. doi: 10.1093/nar/gkv372 (PMC4446432; doi:10.1093/nar/gkv372)
Supplement: SUPPLEMENTARY DATA [file supp_gkv372_nar-03315-d-2014-File009.pdf]

Supporting Data:

Figure *S*1. The N-tails of histones contribute to UV sensitivity. (*A*) WT and histone tail deletions were serially spotted onto YPD and then exposed to varying doses of UV radiation. (*B*) Survival assay of the tailless histone mutants from (*A*).

Figure *S*2. The tH2A:tH3 mutant is less proficient than WT in repairing UV-induced lesions. Cells were arrested in nocodazole and then exposed to 100 J/m^2 of UV and allowed to recover in YPD + nocodazole. Representative alkaline gels for genomic NER show the DNA samples before and after UV exposure in the presence or absence of T_4 endonuclease, which is specific to cyclobutane pyrimidine dimers (CPDs). Quantification of the gels is shown in the graph, and the table indicates the initial CPDs/kb.

Figure *S*3. Protein levels of Mag1 but not Apn1 are downregulated in the tH2A:tH3 mutant after 1 hr MMS treatment. *(A)* Myc-tagged Mag1 in WT and the tailless mutants is analyzed via western blot before and 60 minutes after 0.1% MMS treatment for 10 minutes. *(B)* The same as *(A)* but analyzing myc-tagged Apn1 instead. GAPDH is used as loading control for both blots.

Figure *S*4. Deletion of the N-tails of H2A and H3 decreases Mgt1 protein levels. Western blot analysis of Mgt1 levels in WT and the tailless mutants with GAPDH serving as a loading control.

Figure *S*5*.* Northern blot analysis of *MAG1* mRNA in WT and tH2A:tH3 cells that are overexpressing *MAG1*. Cells were either not treated with MMS or treated with 0.2% MMS for 10 minutes and allowed to recover for 30 minutes. Short and long exposures of *MAG1* are shown and the blot was reprobed for *RDN18*, which serves as the loading control.

Figure *S*6. Western blots of Rad53 phosphorylation after MMS exposure in the tailless histone mutants. *(A)* Cells were in the absence or the presence of 0.1% MMS for 1 hr. *(B)* Time course of WT and the tH3 mutant with continuous exposure to 0.1% MMS.

Figure *S*7. The tH3 mutant is epistatic to *RAD9*. *(A)* WT, tH2A:tH3, and *rad9Δ* derivatives were serially diluted on the YPD plates containing the indicated amounts of MMS. *(B)* Survival assay of WT, the tH3 mutant, and their *rad9Δ* derivatives.

Figure *S*8. tH2A and tH3 epistasis analysis in *(A) rad24Δ*, *(B) mms2Δ, (C)* and *rad30Δ* & *rev1Δ*. *(D)* Survival assay to verify that the N-tail of H3 is not epistatic to *rad30*.

Figure *S*9. tH2A:tH3 is synthetically lethal with *rad6Δ*. The cells on the sc-ura,-leu plate carries both the WT histones (*URA3* plasmid) and the mutant histones (*LEU2* plasmid) in a *rad6Δ* genetic background. The 5-foa plate evicts the shuffle plasmid that contain the WT histones, therefore maintaining the *LEU2* histone plasmid as shown in the diagram.

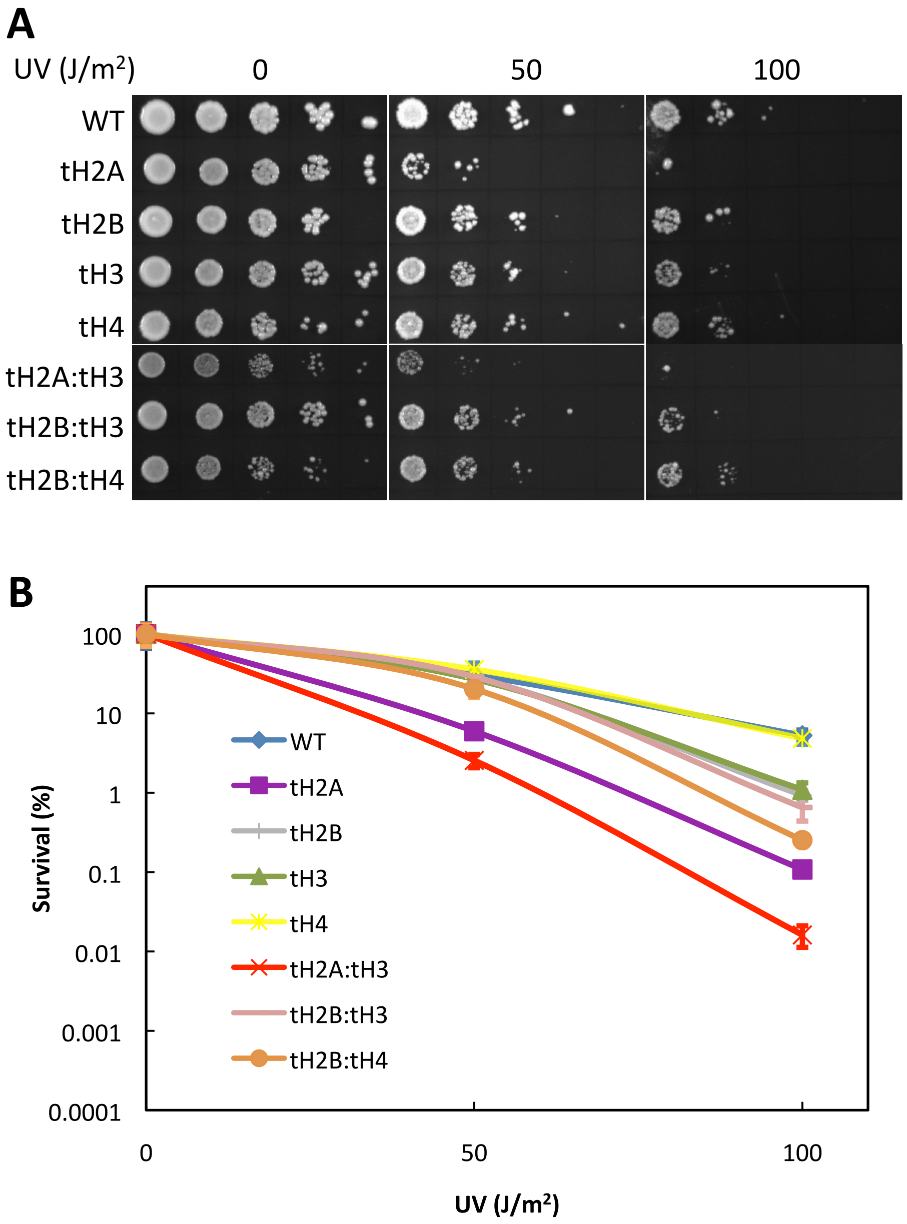
A
UV (J/m²)
0
50
100
WT
tH2A
tH2B
tH3
tH4
tH2A:tH3
tH2B:tH3
tH2B:tH4
B
Survival (%)
100
10
1
0.1
0.01
0.001
0.0001
WT
tH2A
tH2B
tH3
tH4
tH2A:tH3
tH2B:tH3
tH2B:tH4
0
50
100
UV (J/m²)

Figure *S*1

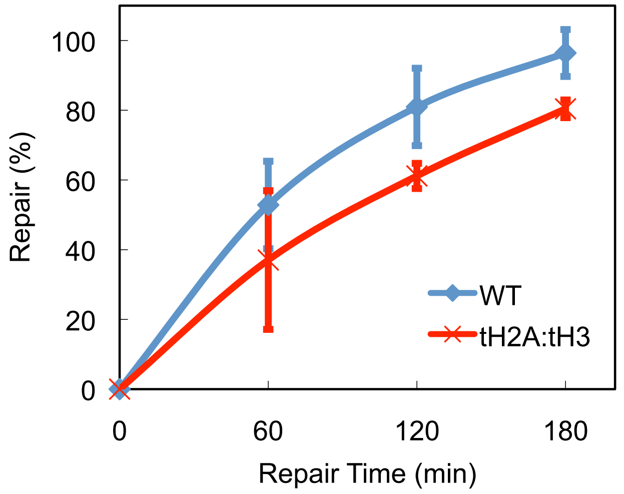

Initial CPDs/kb		
	**Mean**	**Stnd dev.**
**WT**	0.51	0.14
**tH2A:tH3**	0.43	0.08

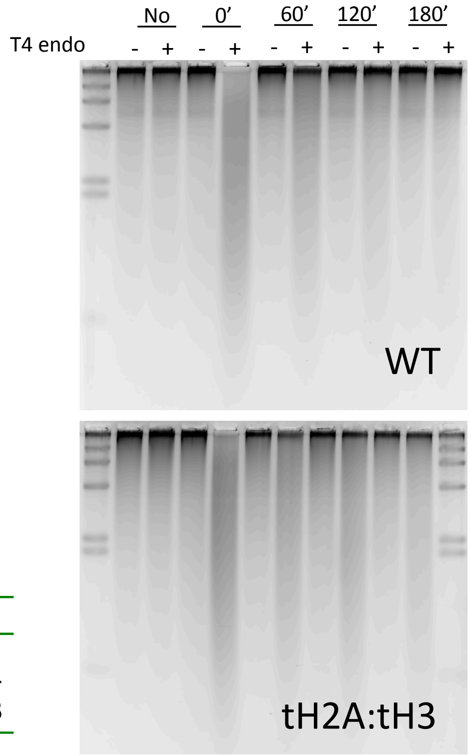

Figure *S*2

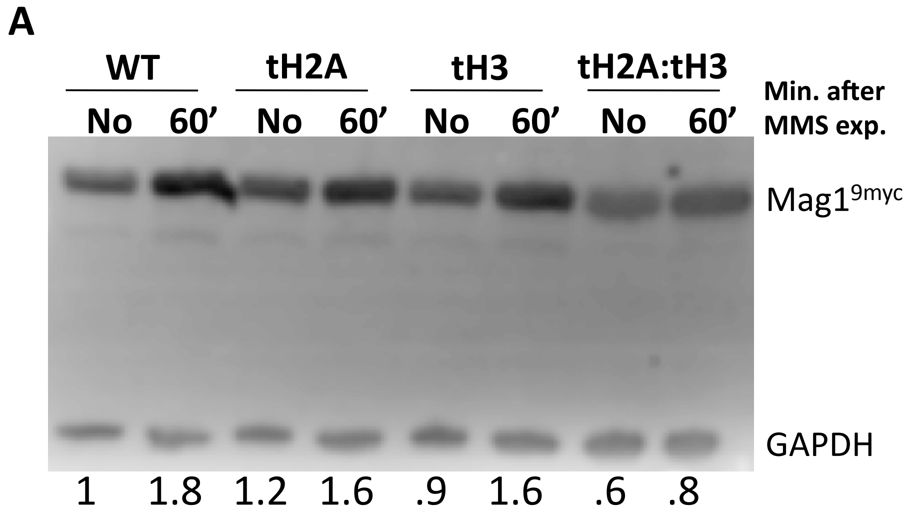
A
WT
tH2A
tH3
tH2A:tH3
Min. after MMS exp.
No 60’ No 60’ No 60’ No 60’
Mag1^{9myc}
GAPDH
1 1.8 1.2 1.6 .9 1.6 .6 .8

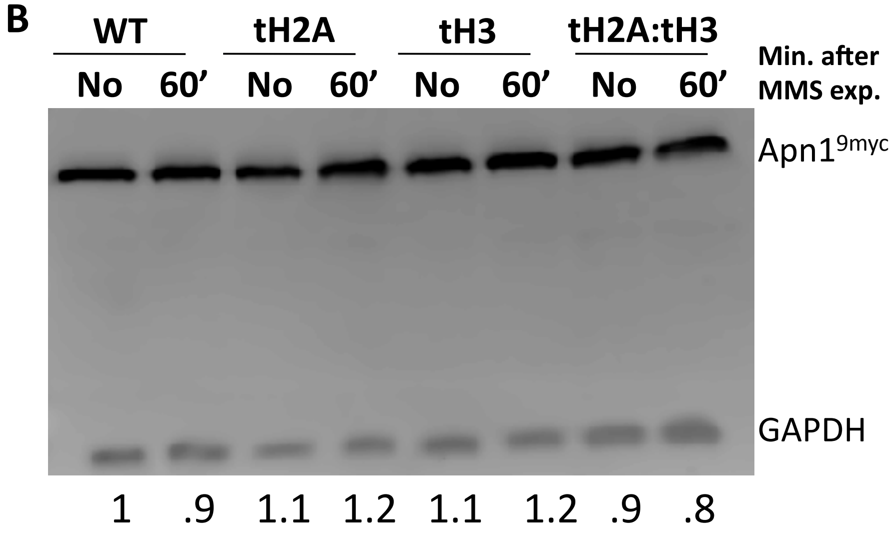
B
WT
tH2A
tH3
tH2A:tH3
Min. after MMS exp.
No 60’ No 60’ No 60’ No 60’
Apn1^{9myc}
GAPDH
1 .9 1.1 1.2 1.1 1.2 .9 .8

Figure *S*3

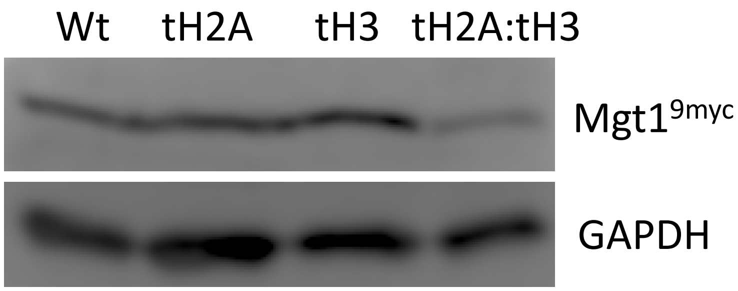

Figure *S*4

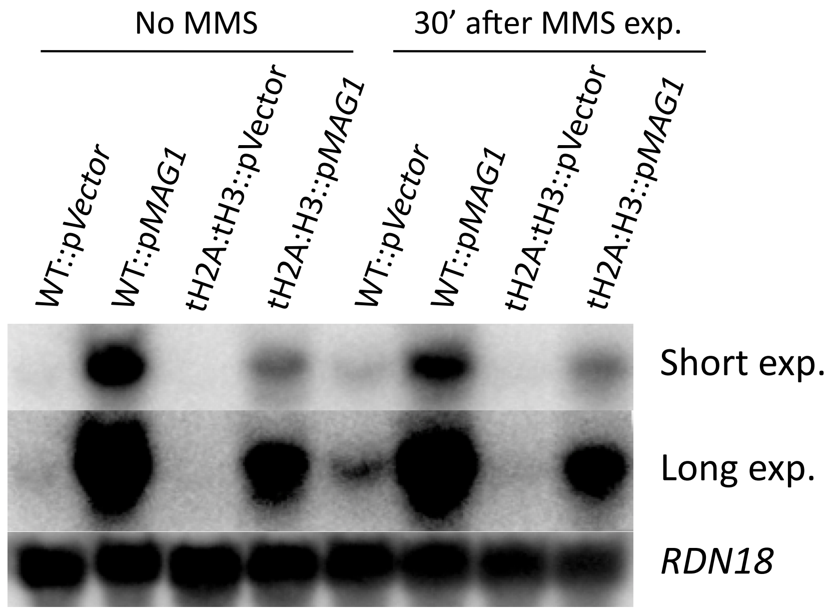
No MMS
30’ after MMS exp.
WT::pVector
WT::pMAG1
tH2A:tH3::pVector
tH2A:H3::pMAG1
WT::pVector
WT::pMAG1
tH2A:tH3::pVector
tH2A:H3::pMAG1
Short exp.
Long exp.
RDN18

Figure *S*5

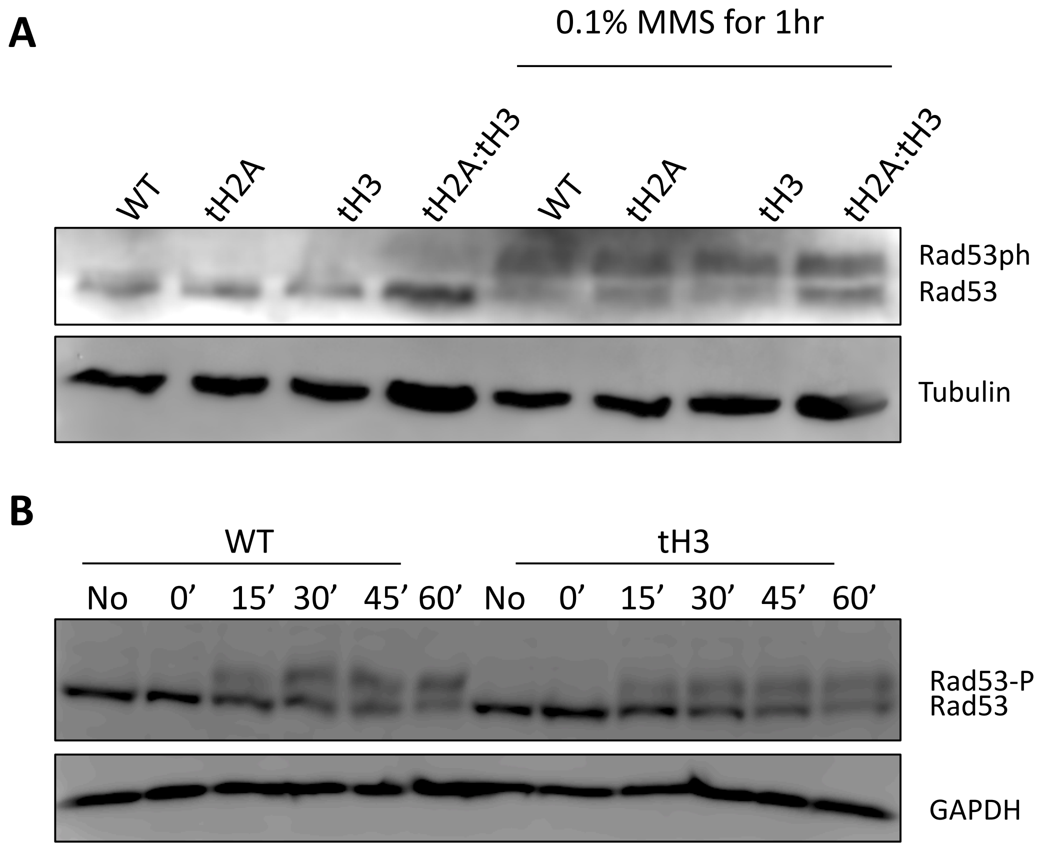
A
0.1% MMS for 1hr
WT
tH2A
tH3
tH2A:tH3
WT
tH2A
tH3
tH2A:tH3
Rad53ph
Rad53
Tubulin
B
WT
tH3
No
0'
15'
30'
45'
60'
No
0'
15'
30'
45'
60'
Rad53-P
Rad53
GAPDH

Figure *S*6

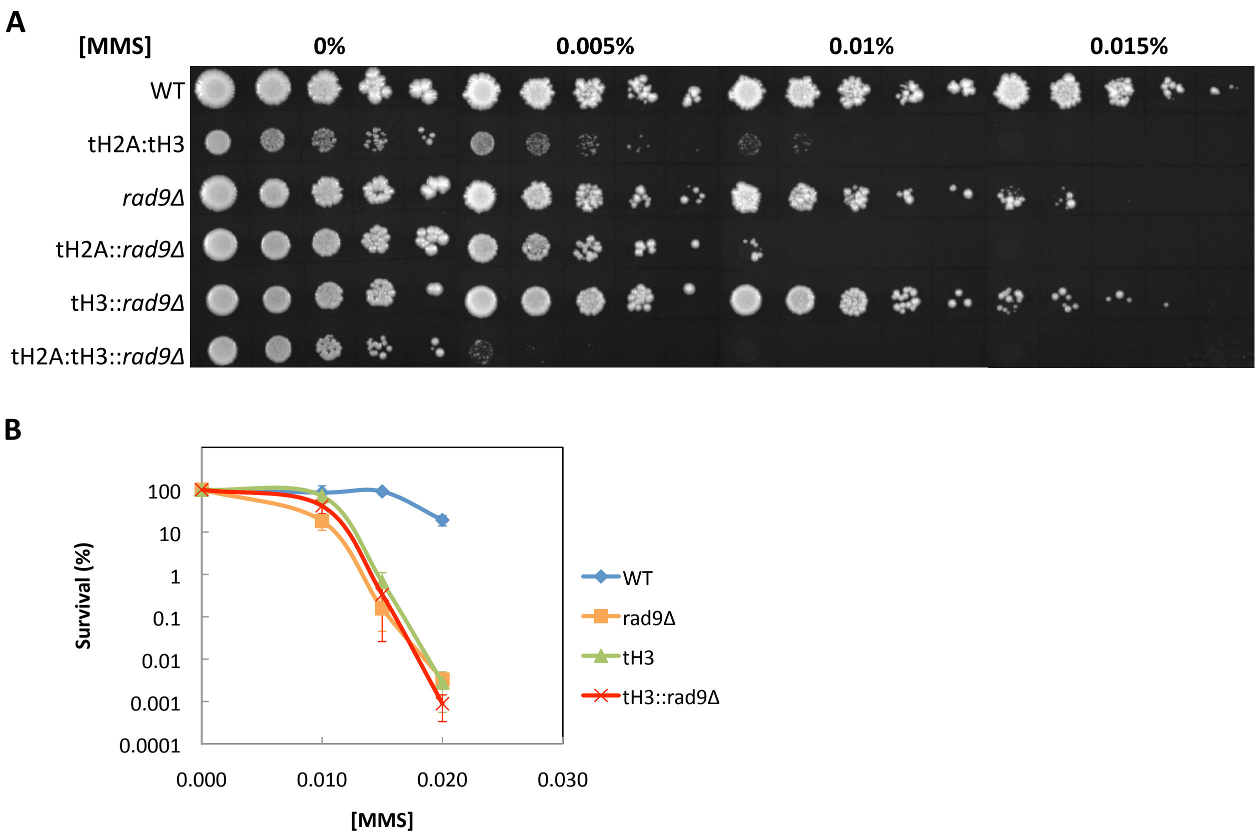

A
[MMS]
0%
0.005%
0.01%
0.015%
WT
tH2A:tH3
rad9Δ
tH2A::rad9Δ
tH3::rad9Δ
tH2A:tH3::rad9Δ
B
Survival (%)
100
10
1
0.1
0.01
0.001
0.0001
0.000
0.010
0.020
0.030
[MMS]
WT
rad9Δ
tH3
tH3::rad9Δ

Figure *S*7

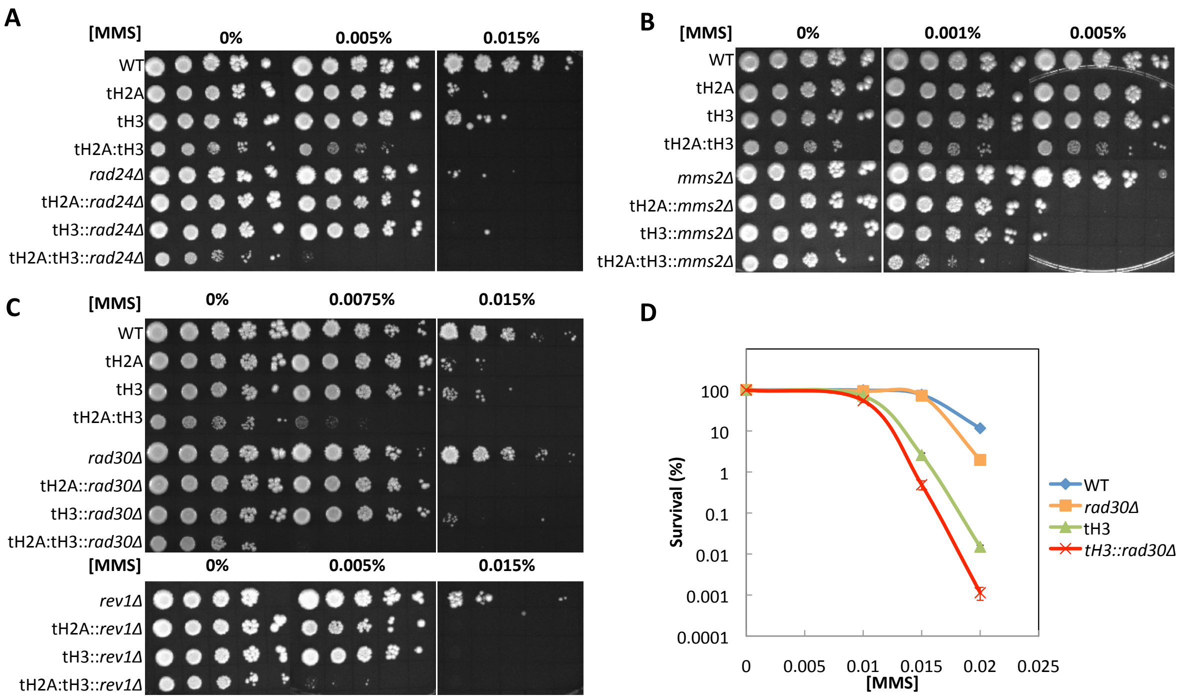

A
[MMS]
0%
0.005%
0.015%
WT
tH2A
tH3
tH2A:tH3
rad24Δ
tH2A::rad24Δ
tH3::rad24Δ
tH2A:tH3::rad24Δ
B
[MMS]
0%
0.001%
0.005%
WT
tH2A
tH3
tH2A:tH3
mms2Δ
tH2A::mms2Δ
tH3::mms2Δ
tH2A:tH3::mms2Δ
C
[MMS]
0%
0.0075%
0.015%
WT
tH2A
tH3
tH2A:tH3
rad30Δ
tH2A::rad30Δ
tH3::rad30Δ
tH2A:tH3::rad30Δ
[MMS]
0%
0.005%
0.015%
rev1Δ
tH2A::rev1Δ
tH3::rev1Δ
tH2A:tH3::rev1Δ
D
Survival (%)
100
10
1
0.1
0.01
0.001
0.0001
0
0.005
0.01
0.015
0.02
0.025
[MMS]
WT
rad30Δ
tH3
tH3::rad30Δ

Figure *S8*

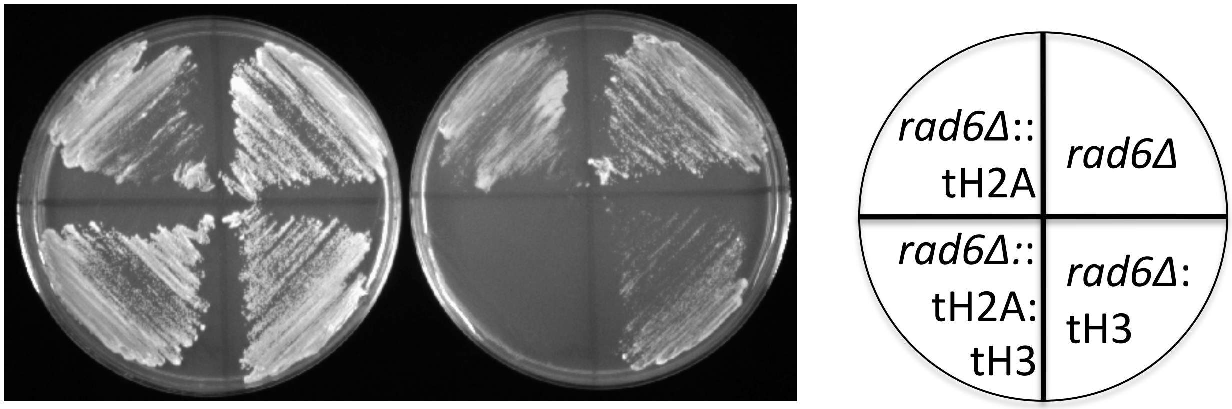

sc-ura,-leu   5-foa

Figure *S*9

Table *S*1.

Strain Name	Genotypes	Used in
RM001[1] (WT)	*MAT**a** his3-1 leu2-0 met15-0 ura3-0 hht1-hhf1::KAN hhf2-hht2::NAT hta1-htb1::HPH hta2-htb2::NAT* p[*CEN LEU2 HTA1-HTB1-HHT2-HHF2*]	(1); Fig. 1-3, 5, 6, *S*1, *S*2, *S*5, S6
RM002 (tH2A)	*MAT**a** his3-1 leu2-0 met15-0 ura3-0 hht1-hhf1::KAN hhf2-hht2::NAT hta1-htb1::HPH hta2-htb2::NAT* p[*CEN LEU2 hta1Δ(1-20)-HTB1-HHT2-HHF2*]	(1); Fig. 1-3, 5, 6, *S*1, *S*5
RM003 (tH2B)	*MAT**a** his3-1 leu2-0 met15-0 ura3-0 hht1-hhf1::KAN hhf2-hht2::NAT hta1-htb1::HPH hta2-htb2::NAT* p[*CEN LEU2 HTA1-htb1Δ(1-32)-HHT2-HHF2*]	(1); Fig. 1, 2, *S*1
RM004 (tH3)	*MAT**a** his3-1 leu2-0 met15-0 ura3-0 hht1-hhf1::KAN hhf2-hht2::NAT hta1-htb1::HPH hta2-htb2::NAT* p[*CEN LEU2 HTA1-HTB1-hht2Δ(1-30)-HHF2*]	(1); Fig. 1-3, 5, 6, *S*1, *S*5
RM005 (tH4)	*MAT**a** his3-1 leu2-0 met15-0 ura3-0 hht1-hhf1::KAN hhf2-hht2::NAT hta1-htb1::HPH hta2-htb2::NAT* p[*CEN LEU2 HTA1-HTB1-HHT2-hhf2Δ(1-16)*]	(1); Fig. 1, 2, *S*1
RM006 (tH2A:tH3)	*MAT**a** his3-1 leu2-0 met15-0 ura3-0 hht1-hhf1::KAN hhf2-hht2::NAT hta1-htb1::HPH hta2-htb2::NAT* p[*CEN LEU2 hta1Δ(1-20)-HTB1-hht2Δ(1-30)-HHF2*]	(1); Fig. 1-3, 5, 6, *S*1, *S*2, *S*5, *S*6
RM007 (tH2B:tH3)	*MAT**a** his3-1 leu2-0 met15-0 ura3-0 hht1-hhf1::KAN hhf2-hht2::NAT hta1-htb1::HPH hta2-htb2::NAT* p[*CEN LEU2 HTA1-htb1Δ(1-32)-hht2Δ(1-30)-HHF2*]	(1); Fig. 1, 2, *S*1
RM008 (tH2B:tH4)	*MAT**a** his3-1 leu2-0 met15-0 ura3-0 hht1-hhf1::KAN hhf2-hht2::NAT hta1-htb1::HPH hta2-htb2::NAT* p[*CEN LEU2 HTA1-htb1Δ(1-32)-HHT2-hhf2Δ(1-16)*]	(1); Fig. 1, *S*1
RM001.1 (*mag1Δ*)	*MAT**a** his3-1 leu2-0 met15-0 ura3-0 mag1Δ::HIS3 hht1-hhf1::KAN hhf2-hht2::NAT hta1-htb1::HPH hta2-htb2::NAT* p[*CEN LEU2 HTA1-HTB1-HHT2-HHF2*]	Fig. 1, 2, 6
RM002.1 (tH2A:: *mag1Δ*)	*MAT**a** his3-1 leu2-0 met15-0 ura3-0 mag1Δ::HIS3 hht1-hhf1::KAN hhf2-hht2::NAT hta1-htb1::HPH hta2-htb2::NAT* p[*CEN LEU2 hta1Δ(1-20)-HTB1-HHT2-HHF2*]	Fig. 6
RM004.1 (tH3:: *mag1Δ*)	*MAT**a** his3-1 leu2-0 met15-0 ura3-0 mag1Δ::HIS3 hht1-hhf1::KAN hhf2-hht2::NAT hta1-htb1::HPH hta2-htb2::NAT* p[*CEN LEU2 HTA1-HTB1-hht2Δ(1-30)-HHF2*]	Fig. 6
RM006.1 (tH2A:tH3:: *mag1Δ*)	*MAT**a** his3-1 leu2-0 met15-0 ura3-0 mag1Δ::HIS3 hht1-hhf1::KAN hhf2-hht2::NAT hta1-htb1::HPH hta2-htb2::NAT* p[*CEN LEU2 hta1Δ(1-20)-HTB1-hht2Δ(1-30)-HHF2*]	Fig. 6
RM001.2 (*rad5Δ*)	*MAT**a** his3-1 leu2-0 met15-0 ura3-0 rad5Δ::HIS3 hht1-hhf1::KAN hhf2-hht2::NAT hta1-htb1::HPH hta2-htb2::NAT* p[*CEN LEU2 HTA1-HTB1-HHT2-HHF2*]	Fig. 6
RM002.2 (tH2A:: *rad5Δ*)	*MAT**a** his3-1 leu2-0 met15-0 ura3-0 rad5Δ::HIS3 hht1-hhf1::KAN hhf2-hht2::NAT hta1-htb1::HPH hta2-htb2::NAT* p[*CEN LEU2 hta1Δ(1-20)-HTB1-HHT2-HHF2*]	Fig. 6
RM004.2 (tH3:: *rad5Δ*)	*MAT**a** his3-1 leu2-0 met15-0 ura3-0 rad5Δ::HIS3 hht1-hhf1::KAN hhf2-hht2::NAT hta1-htb1::HPH hta2-htb2::NAT* p[*CEN LEU2 HTA1-HTB1-hht2Δ(1-30)-HHF2*]	Fig. 6
RM006.2 (tH2A:tH3:: *rad5Δ*)	*MAT**a** his3-1 leu2-0 met15-0 ura3-0 rad5Δ::HIS3 hht1-hhf1::KAN hhf2-hht2::NAT hta1-htb1::HPH hta2-htb2::NAT* p[*CEN LEU2 hta1Δ(1-20)-HTB1-hht2Δ(1-30)-HHF2*]	Fig. 6
RM001.3 (*rad18Δ*)	*MAT**a** his3-1 leu2-0 met15-0 ura3-0 rad18Δ::HIS3 hht1-hhf1::KAN hhf2-hht2::NAT hta1-htb1::HPH hta2-htb2::NAT* p[*CEN LEU2 HTA1-HTB1-HHT2-HHF2*]	Fig. 6
RM002.3	*MAT**a** his3-1 leu2-0 met15-0 ura3-0 rad18Δ::HIS3 hht1-hhf1::KAN hhf2-*	Fig. 6

(tH2A:: *rad18Δ*)	*hht2::NAT hta1-htb1::HPH hta2-htb2::NAT* p[*CEN LEU2 hta1Δ(1-20)-HTB1-HHT2-HHF2*]	
RM004.3 (tH3:: *rad18Δ*)	*MAT***a** *his3-1 leu2-0 met15-0 ura3-0 rad18Δ::HIS3 hht1-hhf1::KAN hhf2-hht2::NAT hta1-htb1::HPH hta2-htb2::NAT* p[*CEN LEU2 HTA1-HTB1-hht2Δ(1-30)-HHF2*]	Fig. 6
RM006.3 (tH2A:tH3:: *rad18Δ*)	*MAT***a** *his3-1 leu2-0 met15-0 ura3-0 rad18Δ::HIS3 hht1-hhf1::KAN hhf2-hht2::NAT hta1-htb1::HPH hta2-htb2::NAT* p[*CEN LEU2 hta1Δ(1-20)-HTB1-hht2Δ(1-30)-HHF2*]	Fig. 6
RM001.4 (*rev3Δ*)	*MAT***a** *his3-1 leu2-0 met15-0 ura3-0 rev3Δ::HIS3 hht1-hhf1::KAN hhf2-hht2::NAT hta1-htb1::HPH hta2-htb2::NAT* p[*CEN LEU2 HTA1-HTB1-HHT2-HHF2*]	Fig. 6
RM002.4 (tH2A:: *rev3Δ*)	*MAT***a** *his3-1 leu2-0 met15-0 ura3-0 rev3Δ::HIS3 hht1-hhf1::KAN hhf2-hht2::NAT hta1-htb1::HPH hta2-htb2::NAT* p[*CEN LEU2 hta1Δ(1-20)-HTB1-HHT2-HHF2*]	Fig. 6
RM004.4 (tH3:: *rev3Δ*)	*MAT***a** *his3-1 leu2-0 met15-0 ura3-0 rev3Δ::HIS3 hht1-hhf1::KAN hhf2-hht2::NAT hta1-htb1::HPH hta2-htb2::NAT* p[*CEN LEU2 HTA1-HTB1-hht2Δ(1-30)-HHF2*]	Fig. 6
RM006.4 (tH2A:tH3:: *rev3Δ*)	*MAT***a** *his3-1 leu2-0 met15-0 ura3-0 rev3Δ::HIS3 hht1-hhf1::KAN hhf2-hht2::NAT hta1-htb1::HPH hta2-htb2::NAT* p[*CEN LEU2 hta1Δ(1-20)-HTB1-hht2Δ(1-30)-HHF2*]	Fig. 6
RM001.10 (*mag1Δ*:: p*Vector*)	*MAT***a** *his3-1 leu2-0 met15-0 ura3-0 mag1Δ::HIS3 hht1-hhf1::KAN hhf2-hht2::NAT hta1-htb1::HPH hta2-htb2::NAT* p[*CEN LEU2 HTA1-HTB1-HHT2-HHF2*] p[*URA3* P_{ADH1}*-Vector*]	Fig. 4
RM006.10 (tH2A:tH3::*mag1Δ*::p*Vector*)	*MAT***a** *his3-1 leu2-0 met15-0 ura3-0 mag1Δ::HIS3 hht1-hhf1::KAN hhf2-hht2::NAT hta1-htb1::HPH hta2-htb2::NAT* p[*CEN LEU2 hta1Δ(1-20)-HTB1-hht2Δ(1-30)-HHF2*] p[*URA3* P_{ADH1}*-Vector*]	Fig. 4
RM001.50 (*mag1Δ*:: p*MAG1*)	*MAT***a** *his3-1 leu2-0 met15-0 ura3-0 mag1Δ::HIS3 hht1-hhf1::KAN hhf2-hht2::NAT hta1-htb1::HPH hta2-htb2::NAT* p[*CEN LEU2 HTA1-HTB1-HHT2-HHF2*] p[*URA3* P_{ADH1}*-MAG1*]	Fig. 4
RM006.11 (tH2A:tH3::*mag1Δ*::p*MAG1*)	*MAT***a** *his3-1 leu2-0 met15-0 ura3-0 mag1Δ::HIS3 hht1-hhf1::KAN hhf2-hht2::NAT hta1-htb1::HPH hta2-htb2::NAT* p[*CEN LEU2 hta1Δ(1-20)-HTB1-hht2Δ(1-30)-HHF2*] p[*URA3* P_{ADH1}*-MAG1*]	Fig. 4
RM001.30 (WT::$MAG1^{9myc}$)	*MAT***a** *his3-1 leu2-0 met15-0 ura3-0 trp1Δ::HIS3* $MAG1^{9myc}$*::TRP1 hht1-hhf1::KAN hhf2-hht2::NAT hta1-htb1::HPH hta2-htb2::NAT* p[*CEN LEU2 HTA1-HTB1-HHT2-HHF2*]	Fig.3, *S*3
RM002.30 (tH2A::$MAG1^{9myc}$)	*MAT***a** *his3-1 leu2-0 met15-0 ura3-0 trp1Δ::HIS3* $MAG1^{9myc}$*::TRP1 hht1-hhf1::KAN hhf2-hht2::NAT hta1-htb1::HPH hta2-htb2::NAT* p[*CEN LEU2 hta1Δ(1-20)-HTB1-HHT2-HHF2*]	Fig.3, *S*3
RM004.30 (tH3::$MAG1^{9myc}$)	*MAT***a** *his3-1 leu2-0 met15-0 ura3-0 trp1Δ::HIS3* $MAG1^{9myc}$*::TRP1 hht1-hhf1::KAN hhf2-hht2::NAT hta1-htb1::HPH hta2-htb2::NAT* p[*CEN LEU2 HTA1-HTB1-hht2Δ(1-30)-HHF2*]	Fig.3, *S*3
RM006.30 (tH2A:tH3:$MAG1^{9myc}$)	*MAT***a** *his3-1 leu2-0 met15-0 ura3-0 trp1Δ::HIS3* $MAG1^{9myc}$*::TRP1 hht1-hhf1::KAN hhf2-hht2::NAT hta1-htb1::HPH hta2-htb2::NAT* p[*CEN LEU2 hta1Δ(1-20)-HTB1-hht2Δ(1-30)-HHF2*]	Fig.3, *S*3
RM001.31 (WT::$POL30^{9myc}$)	*MAT***a** *his3-1 leu2-0 met15-0 ura3-0 trp1Δ::HIS3* $POL30^{9myc}$*::TRP1 hht1-hhf1::KAN hhf2-hht2::NAT hta1-htb1::HPH hta2-htb2::NAT* p[*CEN LEU2 HTA1-HTB1-HHT2-HHF2*]	Fig.6
RM002.31 (tH2A::$POL30^{9myc}$)	*MAT***a** *his3-1 leu2-0 met15-0 ura3-0 trp1Δ::HIS3* $POL30^{9myc}$*::TRP1 hht1-hhf1::KAN hhf2-hht2::NAT hta1-htb1::HPH hta2-htb2::NAT* p[*CEN LEU2 hta1Δ(1-20)-HTB1-HHT2-HHF2*]	Fig.6

RM004.31 (tH3::*POL30*^{9myc})	*MAT***a** *his3-1 leu2-0 met15-0 ura3-0 trp1Δ::HIS3 POL30*^{9myc}*::TRP1 hht1-hhf1::KAN hhf2-hht2::NAT hta1-htb1::HPH hta2-htb2::NAT* p[*CEN LEU2 HTA1-HTB1-hht2Δ(1-30)-HHF2*]	Fig. 6
RM006.31 (tH2A:tH3:*POL30*^{9myc})	*MAT***a** *his3-1 leu2-0 met15-0 ura3-0 trp1Δ::HIS3 POL30*^{9myc}*::TRP1 hht1-hhf1::KAN hhf2-hht2::NAT hta1-htb1::HPH hta2-htb2::NAT* p[*CEN LEU2 hta1Δ(1-20)-HTB1-hht2Δ(1-30)-HHF2*]	Fig. 6

[1]The strain was originally listed as *met15-0*, but we observe growth in the absence of methionine

Reference:

1. Kim, J.A., Hsu, J.Y., Smith, M.M. and Allis, C.D. (2012) Mutagenesis of pairwise combinations of histone amino-terminal tails reveals functional redundancy in budding yeast. *Proceedings of the National Academy of Sciences of the United States of America*, **109**, 5779-5784.
